# Evolution of selenoproteins in the metazoan

**DOI:** 10.1186/1471-2164-13-446

**Published:** 2012-09-03

**Authors:** Liang Jiang, Jiazuan Ni, Qiong Liu

**Affiliations:** 1 College of Life Sciences, Shenzhen University, Shenzhen, 518060, Guangdong Province, PR, China; 2 College of Optoelectronic Engineering, Shenzhen University, Shenzhen, 518060, Guangdong Province PR, China

**Keywords:** Selenoprotein, Selenocysteine, Metazoan, Evolution, Bioinformatics

## Abstract

**Background:**

The selenocysteine (Sec) containing proteins, selenoproteins, are an important group of proteins present throughout all 3 kingdoms of life. With the rapid progression of selenoprotein research in the post-genomic era, application of bioinformatics methods to the identification of selenoproteins in newly sequenced species has become increasingly important. Although selenoproteins in human and other vertebrates have been investigated, studies of primitive invertebrate selenoproteomes are rarely reported outside of insects and nematodes.

**Result:**

A more integrated view of selenoprotein evolution was constructed using several representative species from different evolutionary eras. Using a SelGenAmic-based selenoprotein identification method, 178 selenoprotein genes were identified in 6 invertebrates: *Amphimedon queenslandica, Trichoplax adhaerens, Nematostella vectensis, Lottia gigantean, Capitella teleta*, and *Branchiostoma floridae*. Amphioxus was found to have the most abundant and variant selenoproteins of any animal currently characterized, including a special selenoprotein P (SelP) possessing 3 repeated Trx-like domains and Sec residues in the N-terminal and 2 Sec residues in the C-terminal. This gene structure suggests the existence of two different strategies for extension of Sec numbers in SelP for the preservation and transportation of selenium. In addition, novel eukaryotic AphC-like selenoproteins were identified in sponges.

**Conclusion:**

Comparison of various animal species suggests that even the most primitive animals possess a selenoproteome range and variety similar to humans. During evolutionary history, only a few new selenoproteins have emerged and few were lost. Furthermore, the massive loss of selenoproteins in nematodes and insects likely occurred independently in isolated partial evolutionary branches.

## Background

Selenium is an essential microelement, and selenium deficiency is related to a multitude of diseases and physiological dysfunctions. *In vivo*, Selenium is primarily present in a group of proteins called selenoproteins. Glutathione peroxidase (Gpx), thioredoxin reductase (TR), and iodothyronine deiodinase (DI) are several important selenoproteins that have been thoroughly documented, though the functions of many other newly characterized selenoproteins remain undocumented. The 21^st^ amino acid, a selenocysteine (Sec) residue, is characteristic of all selenoproteins. Notably, the Sec residue is coded by the TGA codon, which is traditionally known as a stop codon [1]. In order to translate the TGA codon into Sec instead of a terminal signal during translation, a specific synthesis complex consisting of several *trans*- factors is enacted in selenoprotein-containing organisms. Accordingly, an RNA structure called the Sec insertion sequence (SECIS) element in the mRNA of selenoproteins recognizes the selenoprotein synthesis complex. The secondary structure of SECIS elements is conservative in selenoproteins genes [2-5].

The complex Sec insertion mechanism makes the expression of selenoproteins *in vitro* very difficult, thus creating technical barriers that have slowed selenoprotein research due to inefficient laboratory methods. In the post-genomic era, the introduction of bioinformatics methods has been advantageous to the study of selenoproteins, resulting in a surge of recent works focusing on the integration of the selenoproteomes of one or more species rather than only a single selenoprotein. Through bioinformatic analysis, the entire human selenoproteome was obtained, providing a complete view of this special protein group [6]. This data forms a comprehensive informational tool for further functional selenoproteome studies.

Consequently, many new organisms have been investigated for the presence and activity of their selenoproteomes, resulting in a myriad of information that still provides only a vague and fragmented view of the distribution and evolution of selenoproteins in living organisms. Contemporary research has revealed selenoprotein in numerous prokaryotic, unicellular algae, and protozoa species [7-12]. Furthermore, similar animal studies using insects, nematodes, and vertebrates has also been reported [13,14]. A comprehensive survey of vertebrate and mammal selenoproteomes was reported recently, depicting the evolution of selenoproteins in vertebrate phyla and providing a wealth of information pertaining to vertebrate selenoproteins characteristics [15]. The selenoproteomes of many other organisms, however, remain undocumented, especially in the invertebrate phyla. Such documentation of selenoproteomes in primitive multicellular organisms may clarify the evolutionary era of metazoans, enhancing overall understanding of animal evolution.

According to previous reports, the variety and size of selenoproteomes varies dramatically between different evolutionary eras. In the animal phyla alone, most vertebrate selenoproteins are absent in both insects and nematodes [16]. Unknown selenoproteomes in other primitive invertebrates, based on previous research in insects and nematodes, would be expected to have very different characteristics than those of more complex vertebrates, such as humans. It is thus possible that massive selenoprotein losses occurred in large areas of certain animal phyla branches.

To explore this issue, 6 invertebrates representing different eras of animal evolutionary history were selected for selenoproteome investigation in the current work. The 6 organisms, each with a recently sequenced genome, were: *Amphimedon queenslandica, Trichoplax adhaerens, Nematostella vectensis, Lottia gigantea, Capitella teleta*, and *Branchiostoma floridae*. Due to the dual function of the TGA codon in selenoprotein genes, regular gene annotation programs failed to correctly predict selenoprotein genes. Therefore, selenoprotein genes were often mis-annotated or totally lost in annotated protein sets published by most genome projects, including the genomes of these 6 organisms. Thus, a selenoprotein gene identification method was developed for selenoprotein identification in newly released genomes. This method achieved previous success in selenoprotein identification in the marine invertebrate *Ciona intestinalis* (Ci) [17]. The current study utilizes similar methods combined with SECIS search and EST comparison to identify invertebrate selenoproteins. Based on these findings, a more integrated and objective view of the evolutionary history of selenoproteins throughout the animal phylum may be established.

## Results and discussion

### Invertebrate selenoproteomes

A total of 178 selenoprotein genes (including several incomplete genes) were identified in 6 marine invertebrates, as shown in Table 1. The total number of selenoproteins found in marine invertebrates ranged from 22–40, similar to the reported vertebrate selenoprotein distribution. All selenoproteins identified in these invertebrates were members of 21 selenoprotein families (all subfamilies were considered members of a single family, *eg.* DI1, DI2, and DI3 all belong to the DI family). The variety of the selenoproteome of marine invertebrates was similar to that of vertebrates, and only a few selenoprotein families were not common between these two stages of animal evolution.

**Table 1:** Selenoproteins found in invertebrates

	**Aq**	**Ta**	**Nv**	**Lg**	**Ct**	**Bf**
15 Kd selenoprotein (Sel15)	1	1	1	1	1	2
Alkyl hydroperoxide reductase C like protein (AphC.like)	3					
Disulfide bond formation protein A (DsbA)			2	1	1	1
Methionine sulfoxide reductase A (MsrA)	1	1	2		2	1
Selenoprotein H (SelH)		2	1		1	1
Selenoprotein J (SelJ)						1
Selenoprotein K (SelK)	1		1	1	1	1
Selenoprotein L (SelL)	1	1	1		1	1
Selenoprotein M (SelM)	1		1		1	1
Selenoprotein N (SelN)	1		1	1	1	1
Selenoprotein O (SelO)	1	1	1	1	1	1
Selenoprotein P (SelP)				1		4
Selenoprotein R (SelR)	1	1	1	1	2	1
Selenoprotein S (SelS)	1		1			1
Selenoprotein T (SelT)	1	1	1	1	1	1
Selenoprotein U (SelU)	4	3	1	1	1	1
Selenoprotein W (SelW)	1	1	2	3	2	2
Selenophosphate synthetase (Sps)	1	1	1	1	1	1
Thioredoxin reductase (TR)	2	2	3	3	2	2
Glutathione peroxidase (Gpx)	1	2	9	4	3	7
Iodothyronine deiodinase (DI)		11		4	12	9
TOTAL	22	28	30	24	34	40

The numbers indicate how many proteins were identified in each selenoprotein family by organism. Organisms are represented by abbreviations: Aq = *Amphimedon queenslandica*, Ta = *Trichoplax adhaerens*, Nv = *Nematostella vectensis*, Lg = *Lottia gigantea*, Ct = *Capitella teleta*, Bf = *Branchiostoma floridae*. The abbreviated name of each selenoprotein is shown in parentheses.

Additionally, both the quantities of selenoprotein genes and selenoprotein families in amphioxus (*Branchiostoma floridae)* were found to be the largest reported in any animal to date. A total of 40 individual selenoproteins were found in amphioxus, and almost all of the invertebrate selenoprotein families were identified in this organism. The one exception was the novel eukaryotic selenoprotein Aq.AphC.like protein. The Aq.AphC.like protein was only found in sponges, showing low similarity to the prokaryotic AphC proteins, and no homologous proteins were found in any other eukaryotic species. All gene structure and position information is detailed in Additional file 1: Figure S1 and Table S1.

### Novel eukaryotic selenoproteins

The Aq.AphC.like selenoprotein family was identified in the genome of the sponge *Amphimedon queenslandica*, an ancient animal native to the Great Barrier Reef that diverged from other metazoans over 600 million years ago [18]. A domain similar to a thioredoxin fold was detected in this protein family. The local amino acid sequence around the Sec residue of the Aq.AphC.like protein showed local homology with prokaryotic AphC proteins, whose function is removal of endogenous hydrogen peroxides in *E. coli* cells [19]. Most prokaryotic AphC proteins are Cysteine-containing, with only four known to contain Sec residues. Low homology was observed between Aq.AphC.like proteins and prokaryotic AphC in Additional file 1: Figure S2. Therefore, the function of Aq.AphC.like protein cannot be determined solely from prokaryotic AphC. Only the Trx-like domain suggests a redox function in the Aq.AphC.like family.

**Figure 1:**
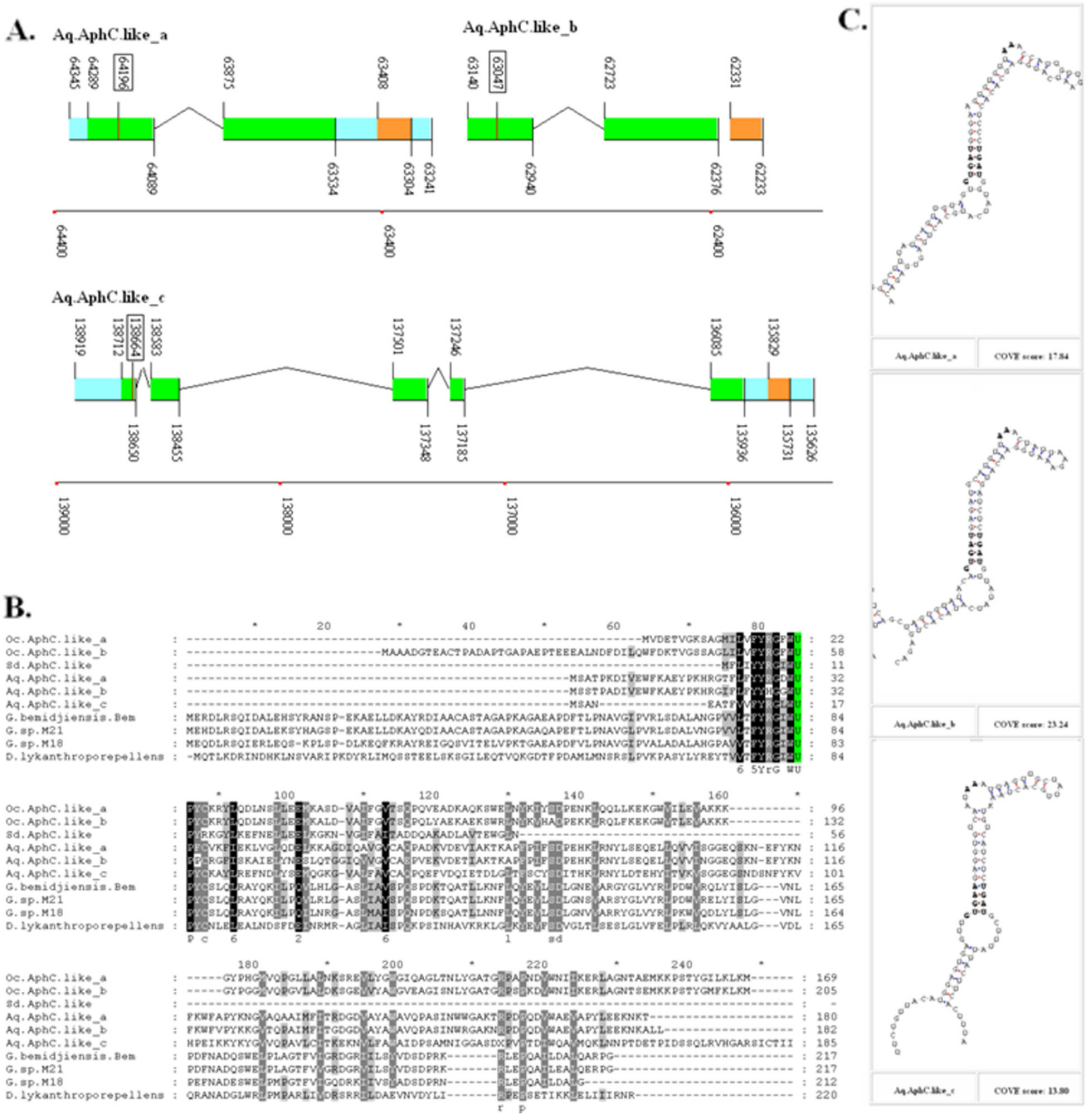
**AphC.like proteins in sponges.A.** The coding regions are indicated by green rectangles, the untranslated regions by blue rectangles, and the SECIS elements by orange rectangles. An intron is indicated by lines connecting the exons. The position of each site in the sequence of the chromosome or scaffold is shown by numbers and bottom coordinates. The position of the Sec-TGA codon is highlighted by the rectangular box around the number. **B.** The multiple alignment of AphC.like proteins and 4 Sec-containing prokaryotic AphC proteins are shown with Sec residues highlighted with a green background. Species names are listed on the left. **C.** The SECIS elements of all Aq.AphC.like genes of *Amphimedon queenslandica* are shown with Cove Scores.

Three Aq.AphC.like proteins were found in the *Amphimedon queenslandica* genome. Two of them were tandemly located in one scaffold, and thus named Aq.AphC.like_a and Aq.AphC.like_b. Both coding regions of Aq.AphC.like_a and Aq.AphC.like_b consist of 2 coding exons. Additionally, the amino acid and SECIS elements are homologous. The third member of this family was found in another scaffold, and thus named Aq.AphC.like_c. Aq.AphC.like_c consists of 5 coding exons. Multiple alignments between Aq.AphC.like proteins and prokaryotic AphC are shown in Figure 1.

No homologous members of the Aq.AphC.like family have been previously reported in eukaryotic selenoproteomes. In order to explore the existence of this family in other species, database including the Nucleotide Collection (nt), Reference Genomic Sequences (refseq_genomic), Whole-genome Shotgun Contigs (wgs), and Expressed Sequence Tags (EST) from the National Center for Biotechnology Information (NCBI) were searched by TblastN, resulting in only 3 hits. Similar positive results were only identified in two other sponge species, *Oscarella carmela* and *Suberites domuncula*. In the *Oscarella carmela*, 2 Oc.AphC.like protein genes can be constructed using ESTs, and the complete coding region and SECIS element can be established (shown in Additional file 1: Figure S3, Figure S4 and Figure S5). A partial amino acid sequence of the Sd.AphC.like protein can be translated from the cDNA sequence of another sponge, *Suberites domuncula*. Though no SECIS information is available due to the incomplete sequencing of this gene, homology analysis shown in Figure 1 provides enough evidence to classify it into this novel family. No other Sec-containing members were found in any other eukaryotic species, suggesting that the Aq.AphC.like proteins belong to a sponge specified selenoprotein family.

Interestingly, all Sec form AphC-containing prokaryotic species are bacteria isolated from highly polluted water [20,21]. The elevated redox activity of Sec compared with that of Cys could be a potential explanation for how such bacteria can survive in severely polluted environments. Sponges reside on the bottom floor of the sea and invariably filter a large volume of seawater, potentially accumulating heavy metals and other contaminants from the environment during their long life-span. The Aq.AphC.like proteins may be critical proteins involved in the protection mechanisms of sponge tissues in response to pollution toxicity [22].

### Selenoproteins lost in vertebrates

In addition to the Aq.AphC.like proteins previously described, the invertebrate phyla contained 2 selenoproteins that were either totally lost or changed into Cys forms. These included disulfide bond formation protein A (DsbA) and methionine sulfoxide reductase A (MsrA). Prior to the investigation of selenoproteins in primitive invertebrates, both DsbA (Sec form) and MsrA (Sec form) were thought to exist only in prokaryotes and unicellular eukaryotes [23]. No DsbA or MsrA in Sec form were found in multicellular animals, such as insects, nematodes, and vertebrates, with the sole exception of DsbA isolated in a sea squirt [17]. In this work, DsbA and MsrA were found to be widespread selenoproteins in sea marine invertebrates, as shown in Table 1.

The Sec/Cys form of DsbA proteins were found in all 6 marine invertebrates in this work. Only the DsbA found in *Amphimedon queenslandica* and *Trichoplax adhaerens* were in the Cys form. As demonstrated by the multiple alignment of DsbA in Additional file 1: Figure S2, these proteins may only be found in prokaryotic, unicellular, plant, fungi, and invertebrate organisms. In higher vertebrates, the DsbA family was completely replaced by other proteins with similar functions, such as protein disulfide isomerase (PDI) or other thioredoxin family members.

MsrA is a member of the methionine sulfoxide reductase (Msr) family. The other members of the Msr family belong to MsrB, in which MsrB1 is also referred to as Selenoprotein R (SelR) [24]. Both MsrA and MsrB sub-families are widespread proteins that can be found in prokaryotes and eukaryotes [25]. During evolutionary history, SelR remained in the Sec form in higher vertebrates. Conversely, all MsrA found in vertebrates was changed to the Cys form. Previously, the Sec form of the MsrA protein was only thought to be present in unicellular microorganisms [7]; however, the current study demonstrates that the Sec form of MsrA also exists in many multicellular invertebrate animals as well. Among the 6 invertebrate marine animals examined, the MsrA protein was only absent in *Lottia gigantea*. As seen in the multiple alignment of MsrA shown in Additional file 1: Figure S2, both the Sec and Cys forms of MsrA were found in the invertebrate phylum.

### Selenoprotein U of invertebrates

Selenoprotein U (SelU) was firstly found in fish and also reported in birds and unicellular eukaryotes, such as *Chlamydomonas reinhardtii*[7,26]. In high mammalian species, such as humans and mice, all SelU proteins exist in Cys form. Three subfamilies of SelU were annotated in humans, SelU1, SelU2 and SelU3. All Sec-containing SelU proteins extracted from the NR database belonged to the SelU1 family, though the function of SelU1 remains unclear. The Prx-like2 structure domain presented in these proteins implies that they belong to the thioredoxin-like superfamily. Many members of the SelU1 family are commonly referred to as C10orf58 or C10orf58-like proteins. Also, numerous homologous SelU2 and SelU3 proteins were annotated in the NR database, though none were observed to be in Sec form. Homologous SelU2 proteins are commonly referred to as C9orf21-like proteins. Homologous SelU3 proteins are commonly referred to as prostamide/prostaglandin F synthase (prFsy) in many species. The prFsy proteins were reported to have a catalytic function in the reduction of prostaglandin-ethanolamide H_2_ (prostamide H_2_) to prostaglandin F (2 alpha) [27].

**Figure 2:**
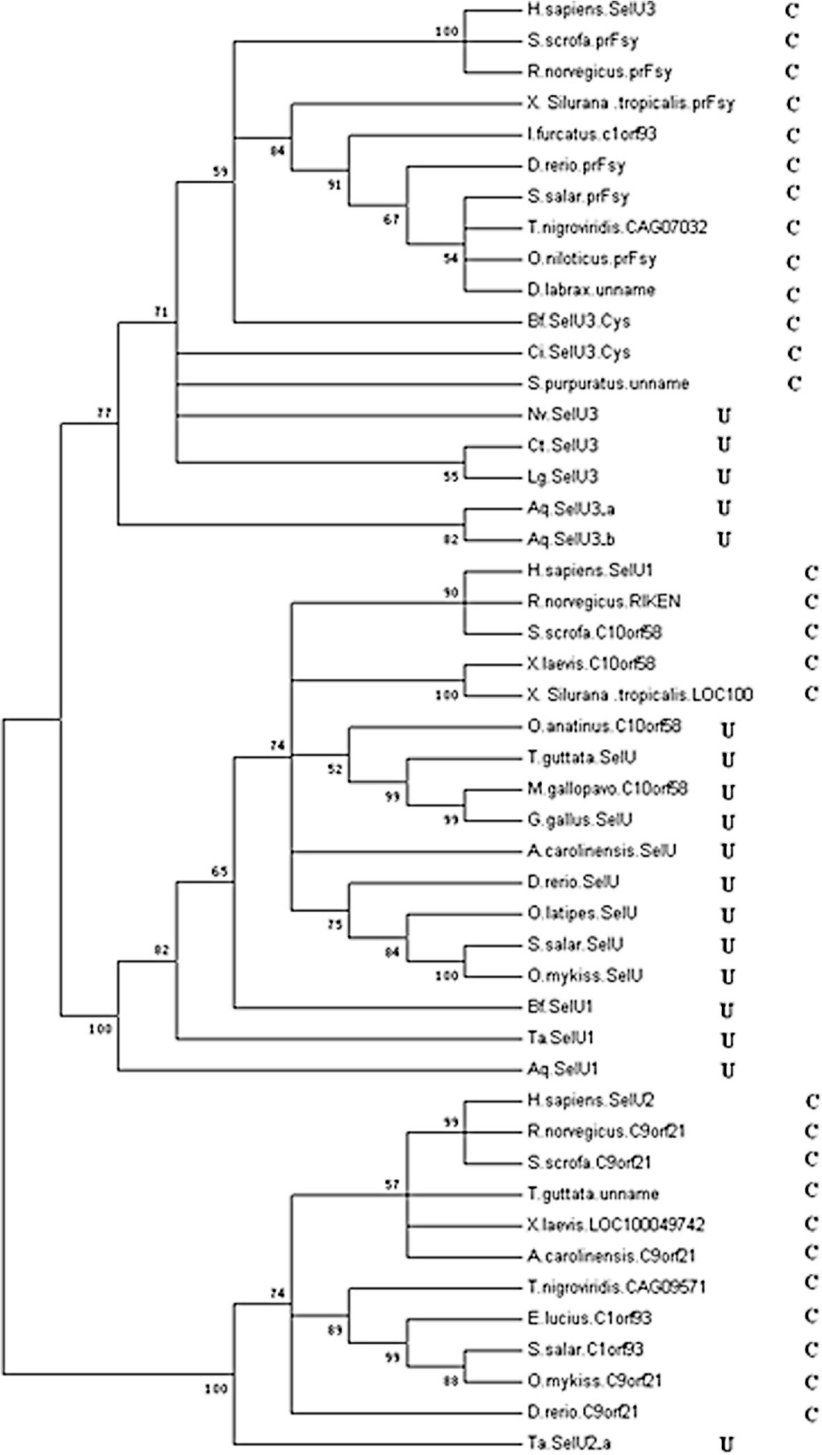
**Phylogenetic tree of eukaryotic metazoan SelU.** Selenoproteins are marked by U, and Cys-form proteins are marked by C. Bootstrap value numbers are shown at each branch point to indicate the reliability of this tree.

**Figure 3:**
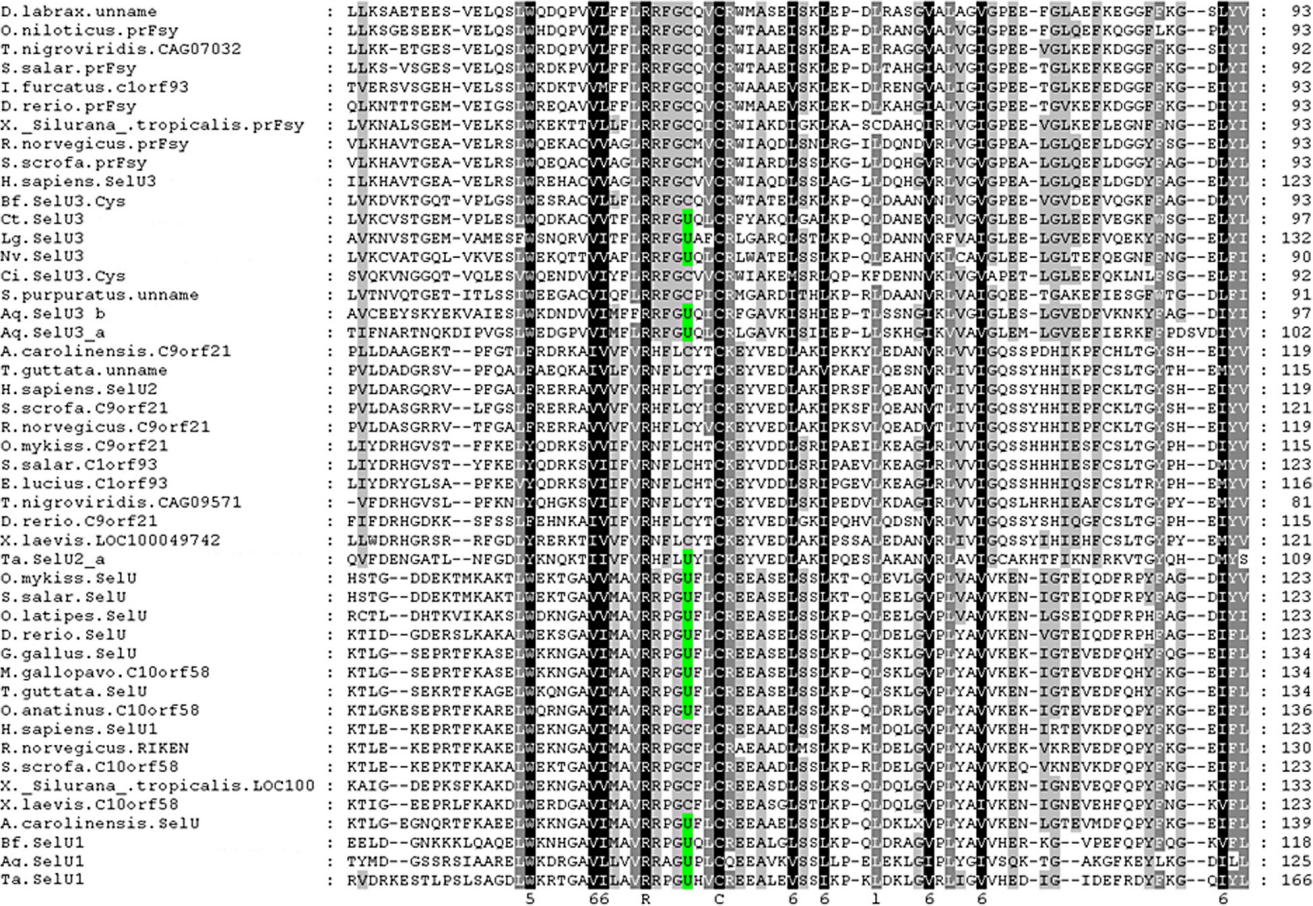
**Multiple alignments of Metazoan SelU proteins.** Sec residues are highlighted with a green background.

More than 10 Sec-containing SelU proteins were found in the 6 invertebrates examined in the present study. Among these, only 3 belonged to the SelU1 protein family, and many more belonged to SelU2 or SelU3 families. Multiple alignment and phylogenic analysis showed that all of these 3 SelU family proteins are widespread and highly conserved in vertebrates, including fishes, amphibians, birds, and mammals (seen in Figure 2 and Figure 3). Additionally, the SelU proteins of invertebrates diverged into 3 groups, classified into different families in accordance with the proteins of their vertebrate descendants. The Sec residues in these proteins were often changed into Cys residues in different stages of these 3 lineages, as shown in the phylogenic tree in Figure 2. In the SelU2 lineage, only one Sec-containing member was found in the primitive invertebrate *Trichoplax adhaerens*, suggesting that Sec to Cys events likely occurred in the early era of invertebrates. The SelU3 lineage represented the most abundant group of invertebrate SelU proteins identified, as 8 SelU3 proteins were found in the 7 species that constituted this group. Interestingly, of these 8 SelU3 proteins, 3 were in Cys form and belonged to more advanced invertebrates, such as sea urchin, amphioxus, and sea squirt. This suggests a clear timeframe during which Sec changed into Cys in the evolution of the SelU3 family. All Cys forms of SelU3 belonged to the *deuterostome* phylum. Thus, the Sec to Cys change event may have occurred before the divergence of this phylum.

In the SelU1 lineage, the Sec to Cys events occurred during much more recent periods. As seen in Figure 2, all invertebrate members of this family as well as many subphyla of vertebrates, including fishes, birds, and reptiles, were shown to contain Sec. Interestingly, not all mammalian forms of SelU1 were Cys form proteins, with the Sec-containing member found in the primitive mammalian platypus, known for retaining its oviparity in a manner similar to birds and reptiles. This information suggests that the Sec to Cys event occurred during the primitive mammalian stage; however, a diverged lineage of Sec to Cys was also found in amphibians. The 2 SelU1 family proteins of two frogs were found to be Cys-form proteins that potentially changed into Cys-form independently, occurring after the divergence of modern amphibians from a common tetrapoda ancestor.

The SelU lineage likely diverged into 3 families before the animal era of evolutionary history began. All 3 families still retain the Sec-form in the progenitors of the animal kingdom, though this form evolved into the Cys-form in higher mammalian species, without exception. Sec to Cys events, however, occurred in different periods of evolutionary history. The widespread presence of all 3 families of SelU in invertebrates serves to construct a more complete and detailed evolutionary map of the SelU protein family in the animal kingdom. It also helps to characterize detailed events, such as the differentiation of diverging lineages and a Sec-losing period for each subfamily.

### Special selenoprotein P

Almost all selenoproteins contain only one Sec residue. Rare selenoprotein families also contain multi-Sec-containing proteins. One of them is the selenoprotein L (SelL) family that contains 2 Sec residues [28]. Other multi-Sec-containing selenoproteins were reported in the selenoprotein W (SelW) family [16]. Interestingly, SelW containing 2 Sec residues was also found in Amphioxus, as shown in Additional file 1: Figure S1.

In the eukaryotic kingdom, selenoprotein P (SelP) is the selenoprotein family that contains the most Sec residues. There are 10 Sec residues in human SelP and up to 17 in that of zebra fish. In human SelP, the Sec residues are distributed in 2 different sections. Only one Sec is located in the N-terminal region that contains a thioredoxin fold domain in SelP. The others are densely located in the C-terminal region. This protein structure is conservative in the whole vertebrate phylum [29]. SelP is considered to play an important role in the preservation and transport of selenium due to an abundance of Sec residues. In mammals, SelP has been reported to be primarily synthesized in the liver, and it is then delivered to the kidney, brain, testes, and other organs [29]. Notably, the hepatic caecum of amphioxus has been suggested to be the origin of the vertebrate liver [30,31], and in this work several SelP proteins were found in the amphioxus genome. SelP was also recently proposed as a biomarker for selenium utilization in humans [29]. Along with the important function reported previously, potential correlation should between Sec numbers of SelP and total numbers of selenoproteins in one organism should be considered [29]. For example, the number of fish selenoproteomes (commonly more than 30 selenoproteins) is generally larger than those of mammals (commonly about 25). Meanwhile, fish SelP generally contains more Sec residues (16–17) than that observed in mammals (7–15) [32].

**Figure 4:**
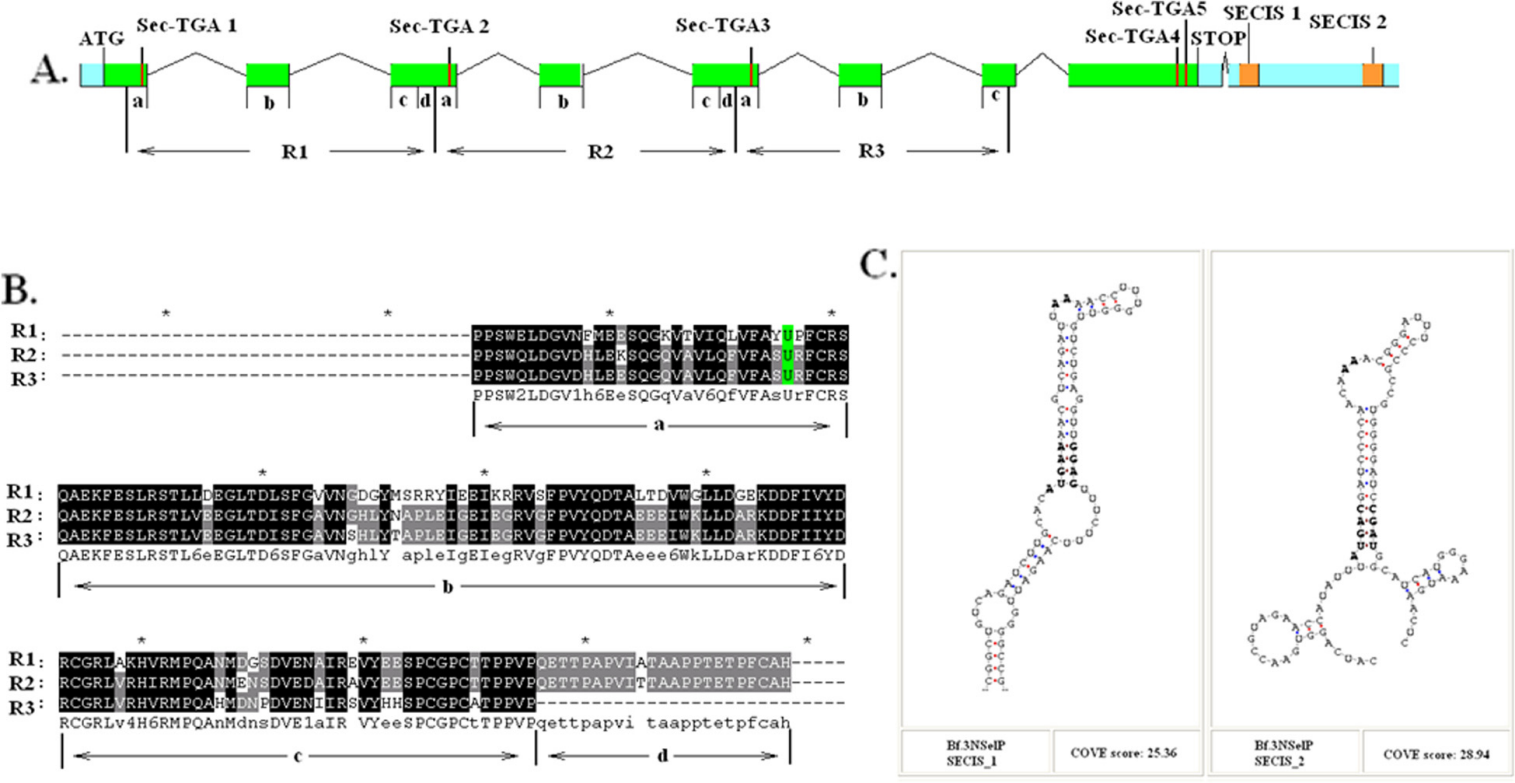
**3NSelP of Amphioxus.A.** The gene structure of 3NSelP with all Sec-TGA codons and SECIS elements is indicated. R1, R2, and R3 represent the 3-repeat regions. Each of the repeated regions can be divided into several parts and labeled parts a, b, c, and d. **B.** Multiple alignment 3-repeat region of the 3NSelP Sec residues and part a, b, c, and d are shown. **C.** The secondary structures of the two SECIS elements of the 3NSelP gene are shown.

SelP is present in all known vertebrate selenoproteomes, but rarely in invertebrates. Only 5 SelP genes were found in this work, and 4 of them were present in amphioxus (*Branchiostoma floridae*). The other one was found in *Lottia gigantea*. Among these, a special SelP was found in amphioxus that contained 5 Sec residues. In this SelP, 3 Sec residues located in the N-terminal region contained 3 repeats of the Trx-like domain. Each was found to be homologous with the N-terminal region containing a Sec residue in vertebrate SelP. The other 2 Sec residues were found in the C-terminal of amphioxus 3NSelP as well as the Sec-rich tail found in vertebrate SelP. This special SelP was named 3NSelP, containing a representative 3 Trx-like domains of the N-terminal. Figure 4 shows that the coding region of 3NSelP consists of 8 exons and that the first 3 Sec residues are located on the 1^st^, 3^rd^, and 5^th^ coding exons. More meticulous manual analysis shows that the 3 Trx-like domains are repeatedly located on the first 7 coding exons. As shown in Figure 4, these 3 repeat regions are indicated as R1, R2 and R3. For each repeat region, the 3 coding exons structures are the same as other vertebrate SelP gene structures previously reported [29]. The multiple alignment of these 3 repeat regions is shown in Figure 4B, demonstrating the strong similarity between these elements. Only short sequence segments in the C-terminal of R1 and R2 do not appear in R3; however, strong similarities are also observed in these 2 short segments. According to multiple alignments and the exon structure of each repeat region, the 3 coding exons of each repeated region were labeled parts a, b, and c. Additionally, the short region missing from R3 is labeled part d in Figure 4A and B. Multiple alignment of R1, R2, and R3 with other amino-terminal vertebrate sequences of SelPs ( Additional file 1: Figure S2) shows that the segment consists of parts a, b, and c homologous with other members, though no similarity appears in part d. Based on these observations, part d was likely developed to conjoin R1, R2, and R3. 

Conserved domain (CD) [33] analysis shows that the complete SelP N domain, a subtype of the Trx-like domain, was found in each of these repeat regions. Previous SelP research reported that the N-terminal region potentially has a redox function. Thus the 3-repeat version of the N-terminal region likely indicates elevated redox activity. Furthermore, preservation and transport roles played by SelP in vertebrates imply that the 3-repeat Sec-containing regions in the SelP of amphioxus may be a method for containing multiple Sec residues, though this would result in low efficiency methods for containing these multiple Sec residues when compared to the dense Sec clustering C-terminal of vertebrate SelP.

It is proposed that two different strategies are possibly applied to increase Sec numbers in the 3NSelP protein. The second way is extending of the sec tail in the C-terminal region. The 2 Sec-TGA are indicated near the C-terminal of this gene, as shown in Figure 4A and Additional file 1: Figure S6. Multiple alignment analysis of SelPs in Additional file 1: Figure S2 shows that no similarity to other C-terminal Sec-rich regions in vertebrates can be detected in this region. The presence of 2 strong SECIS elements located downstream to these TGA codons implies that these two TGA codons are likely read-throughs. Virtually, no TGA codons were found acting as stop codons in any selenoprotein coding genes. Therefore, even without homologues evidence, these 2 TGA codons are likely to be translated into Sec residues in the 3NSelP. The presence of these 2 C-terminal Sec residues provides another way of persevering and transporting multiple Sec residues.

The hypothesis that the C-terminal domain of SelP evolved *de novo* by extension of its C-terminal sequences was proposed by Lobanov *et al.*[29] according to the phenomena that the SelP of *Xenopus* (frogs) is extended by several residues such that their last Sec codons (TGA) correspond to stop signals (TAG/TAA) in other vertebrate SelP genes. Based on this hypothesis, comparison with the 17 Sec residues observed in zebra fish SelP suggests that the presentation of 2 Sec residues in the C-terminal of amphioxus 3NSelP indicates an early stage of extension of Sec numbers in the evolutionary history of SelP.

The N-terminal repeat of 3NSelP is likely caused by DNA duplication and nonreciprocal recombination. Along with repetition of gene domains, the repetition of integrated genes is also a common result of DNA duplication and recombination. Interestingly, another SelP gene was found tandemly located upstream to the 3NSelP gene. This gene is indicated as Bf.SelP_a in the gene structure schematic diagram ( Additional file 1: Figure S1) and multiple alignment ( Additional file 1: Figure S2). Similar exon organization and sequence homology were present in both Bf.SelP_a and 3NSelP. The N-terminal region contains a Trx-like domain and a histidine-rich region that may potentially account for the membrane binding activity observed in both Bf.SelP_a and 3NSelP. Two SECIS elements are also located in the downstream sequence of the Bf.SelP_a gene; however, no C-terminal Sec residue was found in Bf.SelP_a. The gene structure and homology analysis suggested that DNA duplication and recombination produced the repetitive N-terminal regions of 3NSelP and the gene cluster of BfSelP1 and 3NSelP. The mutation of a key position in DNA codons, such as transition from TAG/TAA into TGA to code Sec residues at the C-terminal of 3NSelP, is likely responsible for the divergence between the 2 distinct copies in this cluster.

The presence of this special gene structure and the coexistence of 2 strategies for multiplying Sec residues imply the importance of amphioxus selenoprotein P genes for investigation of the origin and evolution of the SelP family. More functional or systemic research studies pertaining to these clustered SelP genes in amphioxus are necessary before a complete understanding of the profound evolutionary implications of these genes can be formed. Additionally, the clustered and partially repeated SelP genes found in amphioxus may have positive effects on the relative abundance of selenoproteins in this organism, suggesting the role played by SelP in preservation and transport of selenium *in vivo*.

### Gene clusters in invertebrate selenoproteins

**Figure 5:**
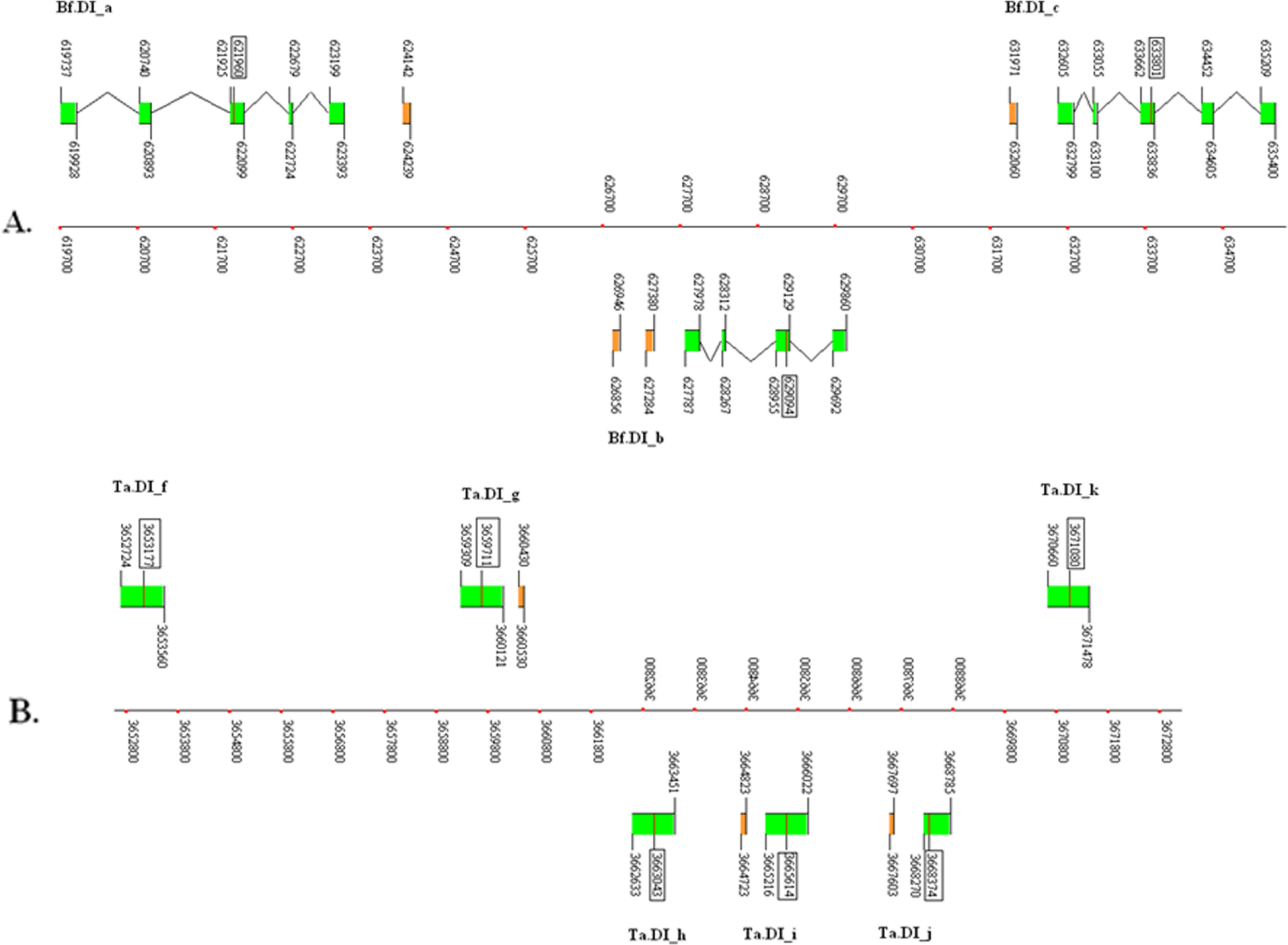
**Gene structures of *Branchiostoma floridae* DIs and * Trichoplax adhaerens* DIs. A.** Gene clusters of Bf.DI_a, Bf.DI_b and Bf.DI_c. The schematic position (under the coordinate) of Bf.DI_b indicates that this gene is on the minus strand. Two strong SECIS elements are located downstream of Bf.DI_b. **B.** Gene cluster of 6 Ta DIs. Ta.DI_h, Ta.DI_i and Ta.DI_j on the minus strand. A strong SECIS element is located downstream of each of Ta.DI_g, Ta.DI_i and Ta.DI_j.

The gene cluster of Bf.SelP_a and 3NSelP was not the only cluster observed in invertebrate selenoproteins. The most significant amount of gene clusters occurred in the iodothyronine deiodinase (DI) family. In the eukaryotic kingdom, almost all DI proteins were found in multicellular animals. Especially in vertebrates, all animals reportedly included selenoproteins containing DI. In the current study, no DI was found in *Amphimedon queenslandica*, and only Cys-form DI genes were found in *Nematostella vectensis*. The clustering duplication of DI was found in *Branchiostoma floridae, Trichoplax adhaerens*, and *Lottia gigantea*. In some of these clusters, 3 or more duplicated genes were tandemly locate in one genome sequence. As seen in Figure 5A, the genes Bf.DI_a, Bf.DI_b, and Bf.DI_c constitute a cluster in which Bf.DI_a and Bf.DI_c are located in the positive strand, while Bf.DI_b is located in the minus strand. Interestingly, 2 strong SECIS elements are located downstream of Bf.DI_b. (Another rare 2 SECIS element containing gene is Nv.Gpx_a found in *Nematostella vectensis*, as shown in Additional file 1: Figure S1 and Figure S3). One of these SECIS elements, however, was not necessary for DI, which possesses only the one TGA codon required for read-through. Because this element appears to serve no current function, future evolutions of this cluster may exhibit loss of the additional SECIS elements. Gene duplication, recombination, and divergence are the main force of genetic evolution [34], and thus clusters consisting of similar genes can be seen as a record of evolutionary events. The 2 SECIS elements of Bf2DI are potentially a result of nonreciprocal recombination during duplication, wherein the DNA sequence including the SECIS elements was copied more times than other sections. The largest gene cluster was found in the *Trichoplax adhaerens*, where 6 DI genes were located tandemly in different strands (Figure 5B). Notably, not all of the genes in this cluster possessed a SECIS element. Only 3 strong SECIS elements were found in the 3 intergenic regions among the middle 4 genes of this cluster. 

Some clusters of invertebrate selenoprotein genes contained incomplete genes or no SECIS genes, such as the cluster of Nv.Gpx_i and Nv.Gpx.h, in which the Nv.Gpx_i does not have SECIS and a complete open reading frame. Similar phenomena can be observed in the clusters (Ct.MsrA_a, Ct.MsrA_b) and (Aq.SelU3_b, Aq.SelU3_c), in which Ct.MsrA_b and Aq.SelU_c are incomplete genes without SECIS elements (Gene structure and location of these cluster can be seen in Additional file 1: Figure S1 and Table S1). Incomplete or absent SECIS sequences imply that these genes are inactive; suggesting that one of the copies of this cluster with the same function has been lost in evolutionary history. These genes report the death phase of the evolution of a certain gene, leaving only an inactive remnant: the pseudogene.

Important evolutionary and divergent information may be found in the gene clusters of invertebrates, such as the beginnings of paralog divergence and their evolutionary termination as pseudogenes. Availability of such information from a wide range of species will provide new ways to explore the evolution of each linage of selenoproteins. More meticulous and concentrated work will certainly be conducted in this area in the future.

### SECIS elements of invertebrates

SECIS elements are essential factors for synthesis of selenoproteins. In eukaryotic selenoprotein mRNAs, the SECIS is located in the 3’ untranslated region (UTR) and appears conserved in the primary and secondary structure. The stem-loop structure consists of 2 helix stems and 2 loops. In most eukaryotic selenoprotein SECIS sequences, a conserved A is located directly preceding the quartet of non-Watson-Crick interacting nucleotides. In combination with the AA in the apical loop, the AUGA_AA sequence may schematically reflect the main conservation of eukaryotic SECIS. In some algae, such as *Chlamydomonas reinhardtii*, *Ostreococcus tauri*, and *Ostreococcus lucimarinus*, most of the SECIS sequences contain the GUGA_AA primary conservation pattern [7,16]. The current work shows that many GUGA_AA patterns in SECIS elements were also found in sponges. Among 21 detected SECIS elements, 12 contained GUGA_AA belonging to the following genes: Aq.AphC.like_a, b, c; Aq.Sel15; Aq.SelK; Aq.SelL; Aq.SelN; Aq.SelT; Aq.SelU3_b; Aq.SPS; and Aq.TR_a; Aq.Gpx; (Shown in Additional file 1: Figure S3). Almost all other SECIS elements of invertebrates, however, are AUGA_AA sequences similar to those observed in vertebrates.

The large numbers of GUGA_AA sequences discovered in such a narrow branch of species, including only several algae and primitive sponges, suggest that the conservation of SECIS elements is more specific to the organism than the selenoprotein family. During selenoprotein synthesis, only a single system was introduced to act with SECIS. SBP2 (SECIS-binding protein 2) may be the protein that combines and reacts with all of the SECIS elements in one organism. Thus, the core pattern of SECIS elements are more conserved in a single organism than in a single selenoprotein family. Moreover, the SBP2 homology between sponge and algae species may have made their GUGA_AA patterns appear more commonly due to their close positions in the evolutionary tree.

### Comparison of metazoan selenoproteins

Selenoproteomes of various species in different branches of the evolutionary tree were identified and analyzed in the post-genomic era. In most primitive organisms, prokaryote and archaea, vast amounts of individual selenoproteins and selenoprotein families were found. A total of 58 selenoprotein families were identified in metagenomic sequences from the Global Ocean Sampling (GOS) [35]; however, the intersection of selenoprotein families in prokaryotes and eukaryotes is small. In fact, only several selenoproteins, such as Gpx, SelW, SPS, DI, MsrA, and DsbA, have been reported in both the prokaryotic and eukaryotic kingdoms [11,16,36]. Thus, during evolutionary history from prokaryotic to eukaryotic stages, the size and content of selenoproteomes have likely undergone extensive changes.

In the eukaryotic stage, the selenoproteomes retained a mosaic in different branches of the evolutionary tree. Especially in unicellular organisms, different amounts and varieties of selenoproteins were reported. The number of selenoprotein families in algae spanned from 0 (red algae, *Cyanidioschyzon merlae*) to 26 (brown algae, *Aureococcus anophagefferens*) [37]. Numerous hypothetical selenoproteins which did not show homology to any other phyla of organism were additionally found in these algae. In another group of unicellular organisms, protozoa, the 4 selenoprotein families, Sel1, Sel2, Sel3, and Sel4, were found in apicomlexa parasites [9]. No homology was found in other species. Reports concerning these algae and protozoa suggested that the selenoproteomes of unicellular organisms are unstable, with a large degree of change occurring in both size and variety of their selenoprotein families.

In the multicellular era, entire loss of selenoproteins occurred in some phyla, such as plants and fungi. In the animal kingdom, stable size and variety are reported in vertebrates. Treating big selenoprotein families, such as Gpx (which includes 8 subfamilies), as a single family, only a few selenoproteins, such as SelU, SelL, and SelJ, are not distributed in all subphyla of vertebrates [38]. However, massive selenoprotein losses were also reported in insects and nematodes, which are both invertebrates [39,40]. Data regarding insects and nematodes implies that the selenoproteomes of invertebrates are still unstable, similar to more primitive unicellular organisms. To provide a more objective view of invertebrate selenoproteomes, the 6 invertebrate species examined in this paper representing different evolutionary stages of invertebrates were selected for further investigation.

**Figure 6:**
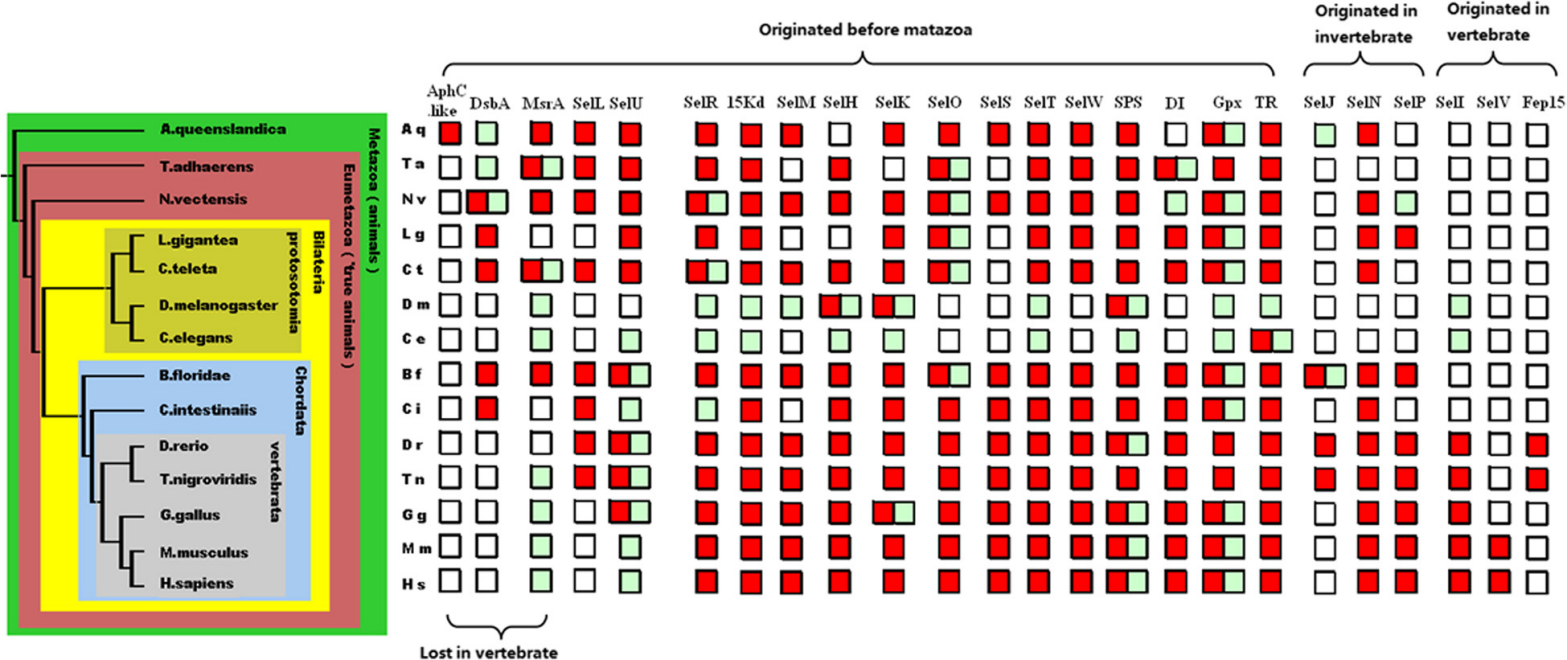
**Selenoproteomes of different animal stages.** The evolutionary roles of animals are shown in the schematic phylogenic tree on the left. All animals are abbreviated by 2 letters indicating their Latin names. The selenoprotein families are presented on the top. The red box indicates the existence of a certain family of selenoproteins in an organism. The green box indicates the existence of Cys-form proteins. The blank box indicates that neither selenoprotein nor Cys-form proteins of this family are detected.

Figure 6 shows selenoproteins of different stages of the animal kingdom, including invertebrates and vertebrates. The schematic phylogenic tree was built based on the phylogeny analysis reported in several genomic research studies of primitive invertebrates, including *Amphimedon queenslandica*, *Trichoplax adhaerens*, and *Nematostella vectensis*[18,41,42]. Seen from the phylogenic tree, the poriferan *Amphimedon queenslandica* is considered the oldest surviving metazoan, representing the most primitive features of multicellular animals. The placozoan *Trichoplax adhaerens* and the cnidarian *Nematostella vectensis* are more evolved animals than sponges, but still very primitive. They are considered the oldest eumetazoan. A more advanced evolutionary stage of the animal kingdom is the bilaterian, with bilateral symmetry. Insects and nematodes belong to a branch of bilaterian named protosotomia. Two other invertebrates, the mollusk *Lottia gigantea* and the annelid *Capitella teleta*, analyzed in this work also belong to this phylum. Vertebrates, including humans, are in the phylum *deuterostomia*. The cephalochordate *Branchiostoma floridae*, the urochordate *Ciona intestinaiis*, and vertebrates constitute the chordate, a subphylum of *deuterostomia*[43,44]. All selenoproteins found in these invertebrates are indicated in Figure 6. Among them are selenoproteins of other reported animals, such as insects, nematodes, and several vertebrates, including fishes, birds, mice, and humans. These are presented in Figure 6 for comparison. 

As seen in Figure 6, the change in variety and size of selenoproteomes of animals from primitive sponges to the most advanced humans are displayed. According the origin time of each selenoprotein family, all animal families can be divided into 3 groups. All selenoprotein families in Group 1 originated in the cellular eukaryotic or prokaryotic era, representing the largest quantity of families. All selenoproteins in Group 2 and Group 3 have not been found in unicellular organisms previously. Selenoproteins of Group 2 were found in invertebrate species, suggesting that Group 2 originated in the invertebrate era. Group 3 originated in the most modern period, the vertebrate era. Only 3 selenoprotein families, SelI (selenoprotein I), Fep15 (fish 15 Kd selenoprotein), and SelV (selenoprotein V), belong to Group 3 [45]. It can be seen that the rare originating events of novel selenoproteins occurred at this point. With the exception of the massive losses in insects and nematodes, only a few selenoproteins, Aq.AphC.like, MsrA, and DsbA, were lost or changed into Cys-forms in the vertebrate stage. Several other selenoproteins, SelJ, SelL, and Fep15, were lost in the tetrapoda stage. The Sec to Cys event occurred in the early period of mammalian history for the SelU lineage. Additionally, only the 2 selenoproteins Aq.AphC.like and Fep15 are specific proteins that only exist in narrow branches (demosponge and fishes).

Apart from several narrowly distributed selenoproteins, most of the selenoprotein families exist in both invertebrates and vertebrates. Even comparisons of primitive sponges to advanced humans indicate that the size and variety of their selenoproteomes are similar. Also, no extensive changes occurred in other intermediate evolutionary stages of invertebrates. Massive loss of selenoproteins was only discovered in insects and nematodes; however, selenoproteomes of mollusk and annelids in the same phylum *protosotomia* with insects and nematodes did not experience massive losses or gains. Therefore, compared to the unstable selenoproteomes of unicellular organisms, the size and variety of multicellular selenoproteomes are much more stable from primitive progenitors to modern humans. Several massive losses occur only as independent events in narrow areas of the evolutionary tree.

The emergence of multicellular animals from unicellular ancestors over 600 million years ago required the evolution of mechanisms for coordinating cell division, growth, specialization, adhesion, and death. From the simple primitive sponge to higher vertebrates, increasing complexity in body plan and organ variety can be observed. Genomic research pertaining to several ancient invertebrates, such as sponge, trichoplax, and sea anemone, indicates that the complexity of their gene sets is similar to vertebrates [41]. Moreover, the selenoproteomes of these primitive invertebrates and other marine invertebrates examined in this paper were also demonstrated to have a similar size and variety as those observed in vertebrates. These findings imply that most of the human selenoprotein families have existed since the earliest era of the animal kingdom. The long period of stable existence of these genes indicates the essential and important role of selenoproteins. Interestingly, a comparison of gene sets of advanced vertebrate animals, primitive sea anemones and other invertebrates, showed extensive loss of genes in insects and nematodes [46,47]. This suggests that the massive selenoprotein loss in these species potentially accompanied reduction of whole gene sets.

## Conclusion

Bioinformatics methods based on the selenoprotein gene assembly algorithm SelGenAmic were used to identify 178 selenoprotein genes from 6 representative species from specific stages of invertebrate evolution. A sponge specific selenoprotein family Aq.AphC.like protein was found in *Amphimedon queenslandica* to be a novel eukaryotic selenoprotein. The two selenoprotein families DsbA and MsrA, previously thought to be only present in unicellular organisms, were found widespread in marine invertebrates. The identification and analysis of SelU1, SelU2, and SelU3 families in invertebrates clarified information about the time of their divergence.

From the cephalochordate animal, amphioxus, that possesses the most abundant and various selenoproteins in the animal kingdom, a special selenoprotein P named 3NselP was found. This selenoprotein is characterized by three Sec residues located in the N-terminal region containing 3-repeat Trx-like domains and two Sec residues located in the C-terminal region. The special gene structure was constructed of 2 different parts containing multiple Sec residues, implying that 2 different strategies for extending the number of Sec residues in selenoprotein P evolved in amphioxus. Another one Sec-containing SelP named as Bf.SelP_a was found located upstream of 3NSelP. The clustering of BfSelP1 and 3NSelP suggests a positive association between abundant selenoproteins in amphioxus. Along with the cluster of SelP genes in amphioxus, several other gene clusters were found in these invertebrates. This information can be translated to a chronological record of events (emerging, diverging, and dying) in the evolution of selenoprotein genes.

Most SECIS elements of sponges are GUGA_AA patterns, which are similar to those found in several green algae. This suggests that the SECIS elements are more conserved by certain species than by gene families, a process most likely associated with the unique selenoprotein synthesis complex system found in each organism.

The selenoproteins obtained in this work support the body of essential information required to produce a more comprehensive and objective view of animal selenoproteomes. Although species with complete genome sequences are currently very rare in the enormous variety of the animal kingdom, the species selected for inclusion in this work are representatives of particular stages of invertebrate evolution. Thus, along with data from other reported species, the selenoproteins examined in this work suggest that the size and variety of selenoproteomes were unstable before the multicellular animal era. In the metazoan phylum, however, the number of selenoproteins and the variety of selenoprotein families vary only slightly from sponge to human, and only a few isolated selenoprotein families were lost or emerged during this period of evolutionary history. Several notable exceptions occurred independently in narrow regions of the evolutionary tree, such as the losses in insects and nematodes, which may be associated with the evolutionary reduction of whole gene sets.

## Methods

### Data resources

The genome sequences and EST sequences used in this work were downloaded from U.S. Department of Energy (DOE) Joint Genome Institute (JGI) and the NCBI database of the U.S. National Library of Medicine. Information including release version number and coverage depth for each organism is shown in Table 2. The organism names were abbreviated as set forth in Table 1. The genome size of these 6 invertebrates is much smaller than the human genome (~3,000 Mbp), which spans from 107 to 522 Mbp. The Bf data was obtained from the assembly v2.0, which is more non-redundant. Therefore, the number of scaffolds is less than that in other invertebrates and the length is longer. The EST sequence sizes and numbers were also shown in Table 2, in which the amount of EST of *Trichoplax adhaerens* was much less than others.

**Table 2:** Summary statistics of data resources

**Organism**	**Release version**	**Coverage depth**	**Genome size (Mbp)**	**Scaffolds numbers**	**EST Size (Mbp)**	**EST numbers**
Aq	v1.0	~8X	143	3,579	42	63,542
Ta	v1.0	~8.1X	107	1,415	8	11,498
Nv	v1.0	~7.8X	356	10,804	133	163,314
Ct	v1.0	~7.9X	334	21,042	119	209,323
Lg	v1.0	~8.9X	360	4,475	203	252,091
Bf	v2.0	~8.1 X	522	398	234	334,502

### General identification procedure

General procedures of our method are described as follows.

(1) Whole genome sequences were scanned to find all TGA codons and other signals including ATG, TAA\TAG, and AG\GT. All exons containing in-frame TGA codons and exons without in-frame TGA were built from these signals. The coding potential of any exon was calculated as the sum of the scores of the signals plus the log-likelihood ratio of a Markov model for coding DNA.

(2) Genes were assembled from exons. For each in-frame TGA containing exon, a best ORF with maximal coding potential score was built with our gene assembly algorithm SelGenAmic.

(3) A search of Sec/Cys pairing and the conservation of its flanking regions was conducted. All genes were translated into amino acid sequences. Local sequences flanking the Sec residue were extracted for detection of similarity in the NCBI non-redundant (nr) protein database by the BLASTp program in order to obtain multiple sequence alignments. Those sequences were screened with conservation in the local regions flanking the Sec residue. Alignments containing Sec/Cys pairing (simplified as U/C pairs), such as the Sec-containing local sequence, had homologous sequences containing Cys residues in the position of Sec in multiple alignments.

(4) Searching against EST databases and EST splicing were conducted. Similarity analysis was performed against EST databases to obtain spliced ESTs. The local DNA sequences flanking the TGA of each gene were searched by BLASTn against the EST database*.*

(5) Checking for SECIS elements was finally conducted to confirm the identified selenoprotein genes.

### Construction of ORFs containing Sec-TGAs

The program Geneid (version 1.2a) [48] was used to obtain common gene signals, such as splice sites, start codon, stop codons, and common potential exons, from genomic sequences. A series of PERL programs were edited to obtain TGA codons from the genome and to build TGA containing exons from common signals and TGA codons. The PERL programs were edited based on the selenoprotein gene assembly algorithm, SelGenAmic, in order to construct all genes containing in-frame TGA codons [17].

### Homology analysis

BLAST programs (version 2.2.18) [49] were obtained from the NCBI ftp server at ftp://ftp.ncbi.nih.gov/blast/db/. The NCBI nr protein database was also downloaded from the NCBI ftp server. All genes containing in-frame TGA codons were searched by the program BLASTp with an E-value cut-off of 1. All similar sequences detected were used to create multiple sequence alignments with ClustalW (version 1.83) [50]. The conservative motif containing the Sec residue of any gene was analyzed by the program using a motif search algorithm, like MAME.

### Search for SECIS elements

RNAfold (version 1.7.2) [51] and PatScan [52] were automatically applied by a PERL program to detect SECIS-like structures from genomic sequences. The SECIS patterns used in the present paper are the same as those used in the search for human SECIS. The COVE scores of SECIS-like structures were evaluated by the online program SECISearch (version 2.19) [6,53].

### Gene structure analysis

EST sequences were downloaded and compared with all predicted selenoprotein genes using the program BLASTn. Highly similar EST sequences were spliced using the SeqMan program from the DNASTAR package http://www.dnastar.com/ and analyzed for selenoprotein gene structure. The constructed genes were homologously compared to genomic sequences with the program Sim4 [54] to find the locations of exons and introns in the genome, shown as position numbers in gene structure figures.

### Phylogenetic analysis

Multiple alignments of amino acid sequences were generated using the ClustalX program (version 1.83) [55]. The unrooted phylogenetic tree with unscaled distance branches was generated using the program MEGA 3.1 http://meme.sdsc.edu/meme4_1/intro.html with the Neighbor-Joining method. Tests for phylogenetic analyses were done by 1000 replications of the Bootstrap algorithm.

## Competing interests

The authors declare that they have no competing interests.

## Authors' contributions

LJ carried out the whole research work including program edit, algorithm design, selenoprotein identification from the genomes, and draft making. QL was responsible for the project design, key-issue discussion and manuscript writing. JN was responsible for the project design, progress and coordination. All authors read and approved the final manuscript.

## Supplementary Material

Additional file 1 **The following additional data are included within the additional file.** The genomic chromosome or scaffold from which the selenoprotein gene was identified in this paper is shown in Supplemental **Table S1.** Multiple alignments of all newly identified selenoproteins and their homologous sequences are shown in Supplemental **Figure S2.** Gene structures of the newly identified selenoprotein genes in marine invertebrates are shown in Supplemental **Figure S1.** The secondary structures and COVE scores of the SECIS elements of these selenoprotein genes are shown in Supplemental **Figure S3.** The DNA sequences and amino acid sequences of Oc.AphC.lile_a and b are shown in Supplemental **Figure S4** and **S5.** The DNA sequence and amino acid sequence of Aq.3NSelP are shown in Supplemental **Figure S6.** Click here for file
