# Supplementary material for: Evolution of selenoproteins in the metazoan
Source: BMC Genomics. 2012 Sep 3;13:446. doi: 10.1186/1471-2164-13-446 (PMC3473315; doi:10.1186/1471-2164-13-446)
Supplement: Additional file 1 — The following additional data are included within the additional file. The genomic chromosome or scaffold from which the selenoprotein gene was identified in this paper is shown in Supplemental Table S1. Multiple alignments of all newly identified selenoproteins and their homologous sequences are shown in Supplemental Figure S2. Gene structures of the newly identified selenoprotein genes in marine invertebrates are shown in Supplemental Figure S1. The secondary structures and COVE scores of the SECIS elements of these selenoprotein genes are shown in Supplemental Figure S3. The DNA sequences and amino acid sequences of Oc.AphC.lile_a and b are shown in Supplemental Figure S4 and S5. The DNA sequence and amino acid sequence of Aq.3NSelP are shown in Supplemental Figure S6. [file 1471-2164-13-446-S1.doc]

**Table S1. The genomic chromosome or scaffold from which the selenoprotein gene was identified.**

| **Aq** |  | **Ta** |  | **Nv** |  | **Lg** |  | **Ct** |  | **Bf** |  |
| --- | --- | --- | --- | --- | --- | --- | --- | --- | --- | --- | --- |
| Aq.Sel15 | (+)GL345182 | Ta.Sel15 | (+)scaffold_2 | Nv.Sel15 | (-)scaffold_49 | Lg.Sel15 | (-)sca_10 | Ct.Sel15 | (-)scaffold_65 | Bf.Sel15_a | (-)Bf_V2_240 |
|  |  |  |  |  |  |  |  |  |  | Bf.Sel15_b | (+)Bf_V2_167 |
| Aq.AphC.like_a | (-)GL345392 |  |  |  |  |  |  |  |  |  |  |
| Aq.AphC.like_b | (-)GL345262 |  |  |  |  |  |  |  |  |  |  |
| Aq.AphC.like_c | (-)GL345392 |  |  |  |  |  |  |  |  |  |  |
|  |  |  |  | Nv.DsbA_a | (-)scaffold_36 | Lg.DsbA | (+)sca_64 | Ct.DsbA | (+)scaffold_136 | Bf.DsbA | (+)Bf_V2_196 |
|  |  |  |  | Nv.DsbA_b | (-)scaffold_36 |  |  |  |  |  |  |
| Aq.MsrA | (+)GL345203 | Ta.MsrA | (+)scaffold_6 | Nv.MsrA_a | (+)scaffold_32 |  |  | Ct.MsrA_a | (-)scaffold_920 | Bf.MsrA | (-)Bf_V2_187 |
|  |  |  |  | Nv.MsrA_b | (+)scaffold_21 |  |  | Ct.MsrA_b | (+)scaffold_17746 |  |  |
|  |  | Ta.SelH_a | (-)scaffold_5 | Nv.SelH | (-)scaffold_140 |  |  | Ct.SelH | (+)scaffold_44 | Bf.SelH | (+)Bf_V2_78 |
|  |  | Ta.SelH_b | (+)scaffold_13 |  |  |  |  |  |  |  |  |
|  |  |  |  |  |  |  |  |  |  | Bf.SelJ | (+)Bf_V2_34 |
| Aq.SelK | (+)GL345188 |  |  | Nv.SelK | (-) scaffold_119 | Lg.SelK | (+)sca_46 | Ct.SelK | (+)scaffold_526 | Bf.SelK | (+)Bf_V2_226 |
| Aq.SelL | (+)GL345222 | Ta.SelL | (-)scaffold_22 | Nv.SelL | (+)scaffold_126 |  |  | Ct.SelL | (-)scaffold_502 | Bf.SelL | (+)Bf_V2_161 |
| Aq.SelM | (+)GL345631 |  |  | Nv.SelM | (+)scaffold_37 |  |  | Ct.SelM | (-)scaffold_229 | Bf.SelM | (+)Bf_V2_142 |
| Aq.SelN | (-)GL345319 |  |  | Nv.SelN | (-)scaffold_89 | Lg.SelN | (-)sca_30 | Ct.SelN | (-)scaffold_606_ | Bf.SelN | (+)Bf_V2_96 |
| Aq.SelO | (-)GL345152 | Ta.SelO | (-)scaffold_2 | Nv.SelO | (-)scaffold_5 | Lg.SelO | (-)sca_61 | Ct.SelO | (-)scaffold_174 | Bf.SelO | (+)Bf_V2_205 |
|  |  |  |  |  |  | Lg.SelP | (+)sca_53 |  |  | Bf.3NSelP | (-)Bf_V2_149 |
|  |  |  |  |  |  |  |  |  |  | Bf.SelP_a | (-)Bf_V2_149 |
|  |  |  |  |  |  |  |  |  |  | Bf.SelP_b | (-)Bf_V2_97 |
|  |  |  |  |  |  |  |  |  |  | Bf.SelP_c | (-)Bf_V2_127 |
| Aq.SelR | (-)GL345193 | Ta.SelR | (-)scaffold_5 | Nv.SelR | (+)scaffold_75 | Lg.SelR | (-)sca_59 | Ct.SelR_a | (+)scaffold_320 | Bf.SelR | (+)Bf_V2_48 |
|  |  |  |  |  |  |  |  | Ct.SelR_b | (+)scaffold_551 |  |  |
| Aq.SelS | (-)GL345316 |  |  | Nv.SelS | (+)scaffold_211 |  |  |  |  | Bf.SelS | (+)Bf_V2_207 |
| Aq.SelT | (-)GL345142 | Ta.SelT | (-)scaffold_11 | Nv.SelT | (-)scaffold_469 | Lg.SelT | (-)sca_33_ | Ct.SelT | (+)scaffold_4 | Bf.SelT | (+)Bf_V2_104 |
| Aq.SelU1 | (+)GL345258 | Ta.SelU1 | (-)scaffold_8 | Nv.SelU3 | (-)scaffold_268 | Lg.SelU3 | (+)sca_71 | Ct.SelU3 | (+)scaffold_247 | Bf.SelU1 | (-)Bf_V2_171 |
| Aq.SelU3_a | (-)GL345161 | Ta.SelU2_a | (-)scaffold_7 |  |  |  |  |  |  |  |  |
| Aq.SelU3_b | (-)GL345405 | Ta.SelU2_b | (-)scaffold_7 |  |  |  |  |  |  |  |  |
| Aq.SelU3_c | (-)GL345405 |  |  |  |  |  |  |  |  |  |  |
| Aq.SelW1 | (+)GL345199 | Ta.SelW2 | (+)scaffold_14 | Nv.SelW1 | (+)scaffold_185 | Lg.SelW1_a | (-)sca_128 | Ct.SelW2_a | (-)scaffold_115 | Bf.SelW1 | (+)Bf_V2_245 |
|  |  |  |  | Nv.SelW2 | (-) scaffold_33 | Lg.SelW1_b | (-)sca_128 | Ct.SelW2_b | (-) scaffold_10 | Bf.Selw2 | (+)Bf_V2_233 |
|  |  |  |  |  |  | Lg.SelW2 | (-) sca_40 |  |  |  |  |
| Aq.SPS | (-)GL345134 | Ta.SPS | (+)scaffold_6 | Nv.SPS | (+)scaffold_43 | Lg.SPS | (-)sca_90 | Ct.SPS | (-)scaffold_170 | Bf.SPS | (+)Bf_V2_140 |
| Aq.TR_a | (-)GL345646 | Ta.TR_a | (+)scaffold_12 | Nv.TR_a | (+)scaffold_124 | Lg.TR_a | (+)sca_1034 | Ct.TR_a | (+) scaffold_296_ | Bf.TR_a | (+)Bf_V2_167 |
| Aq.TR_b | (+)GL345175 | Ta.TR_b | (-)scaffold_1 | Nv.TR_b | (+)scaffold_20 | Lg.TR_b | (-) sca_71 | Ct.TR_b | (-) scaffold_873 | Bf.TR_b | (+) Bf_V2_184 |
|  |  |  |  | Nv.TR_c | (+)scaffold_20 | Lg.TR_c | (+) sca_20 |  |  |  |  |
| Aq.Gpx | (-)GL345360 | Ta.Gpx_a | (-)scaffold_4 | Nv.Gpx_a | (-)scaffold_25 | Lg.Gpx_a | (+) sca_11 | Ct.Gpx_a | (-)scaffold_384 | Bf.Gpx_a | (-)Bf_V2_207 |
|  |  | Ta.Gpx_b | (-)scaffold_9 | Nv.Gpx_b | (+)scaffold_499 | Lg.Gpx_b | (+) sca_5 | Ct.Gpx_b | (-)scaffold_20 | Bf.Gpx_b | (-)Bf_V2_271 |
|  |  |  |  | Nv.Gpx_c | (-)scaffold_4 | Lg.Gpx_c | (-) sca_5 | Ct.Gpx_c | (-)scaffold_10 | Bf.Gpx_c | (-)Bf_V2_271 |
|  |  |  |  | Nv.Gpx_d | (+)scaffold_4 | Lg.Gpx_d | (-) sca_2 |  |  | Bf.Gpx_d | (-)Bf_V2_165 |
|  |  |  |  | Nv.Gpx_e | (+)scaffold_4 |  |  |  |  | Bf.Gpx_e | (-)Bf_V2_165 |
|  |  |  |  | Nv.Gpx_f | (+)scaffold_9 |  |  |  |  | Bf.Gpx_f | (-)Bf_V2_165 |
|  |  |  |  | Nv.Gpx_g | (+)scaffold_52 |  |  |  |  | Bf.Gpx_g | (+) Bf_V2_34 |
|  |  |  |  | Nv.Gpx_h | (-)scaffold_32 |  |  |  |  |  |  |
|  |  |  |  | Nv.Gpx_i | (-)caffold_32 |  |  |  |  |  |  |
|  |  | Ta.DI_a | (-)scaffold_5 |  |  | Lg.DI_a | (+) sca_1 | Ct.DI_a | (+)scaffold_98 | Bf.DI_a | (+)Bf_V2_113 |
|  |  | Ta.DI_b | (+)scaffold_5 |  |  | Lg.DI_b | (-)sca_53 | Ct.DI_b | (-)scaffold_10508 | Bf.DI_b | (-)Bf_V2_113 |
|  |  | Ta.DI_c | (-)scaffold_5 |  |  | Lg.DI_c | (+) sca_53 | Ct.DI_c | (+)scaffold_525 | Bf.DI_c | (-)Bf_V2_113 |
|  |  | Ta.DI_d | (-)scaffold_6 |  |  | Lg.DI_d | (+) sca_53 | Ct.DI_d | (+)scaffold_288 | Bf.DI_d | (+)Bf_V2_135 |
|  |  | Ta.DI_e | (+)scaffold_1003 |  |  |  |  | Ct.DI_e | (+)scaffold_672 | Bf.DI_e | (-)Bf_V2_225 |
|  |  | Ta.DI_f | (+)scaffold_7 |  |  |  |  | Ct.DI_f | (-)scaffold_531 | Bf.DI_f | (-)Bf_V2_104 |
|  |  | Ta.DI_g | (+)scaffold_7 |  |  |  |  | Ct.DI_g | (+)scaffold_1012 | Bf.DI_g | (+)Bf_V2_388 |
|  |  | Ta.DI_h | (-)scaffold_7 |  |  |  |  | Ct.DI_h | (-)scaffold_668 | Bf.DI_h | (-)Bf_V2_21 |
|  |  | Ta.DI_i | (-)scaffold_7 |  |  |  |  | Ct.DI_i | (-)scaffold_9 | Bf.DI_i | (-)Bf_V2_21 |
|  |  | Ta.DI_j | (-)scaffold_7 |  |  |  |  | Ct.DI_j | (-)scaffold_434 |  |  |
|  |  | Ta.DI_k | (+)scaffold_7 |  |  |  |  | Ct.DI_k | (+)scaffold_12 |  |  |
|  |  |  |  |  |  |  |  | Ct.DI_l | (-)scaffold_397 |  |  |

The (+) indicates the selenoprotein gene is on the positive strand. The (-) indicates the gene is on the minus strand.

Sel15


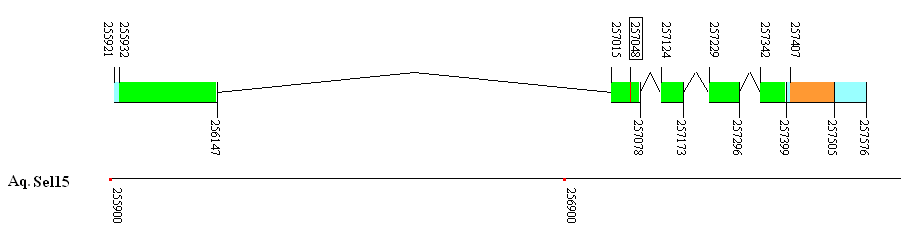

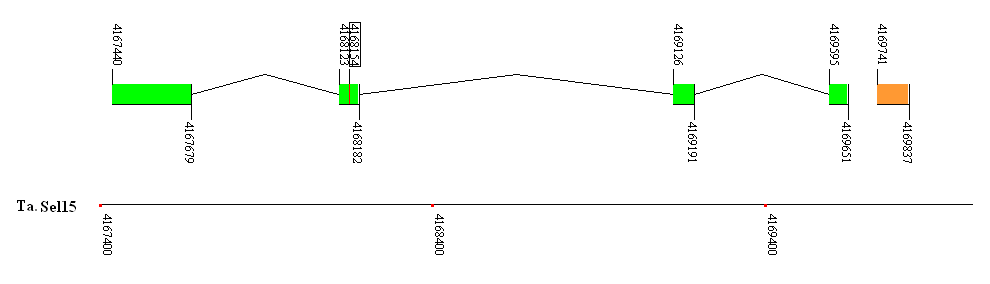


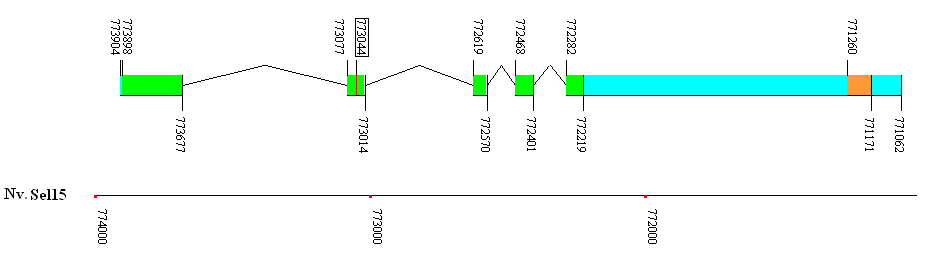


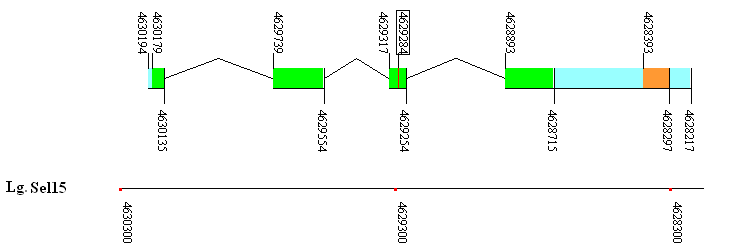


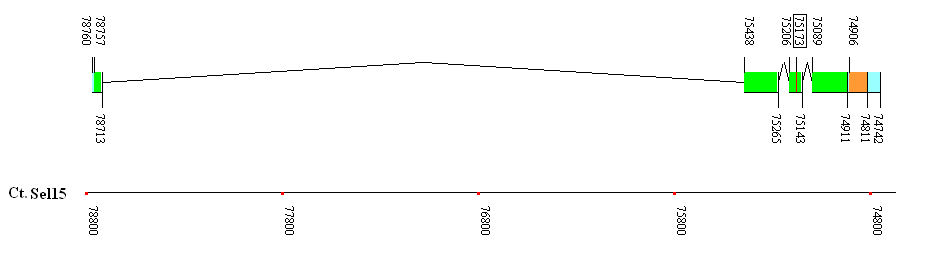

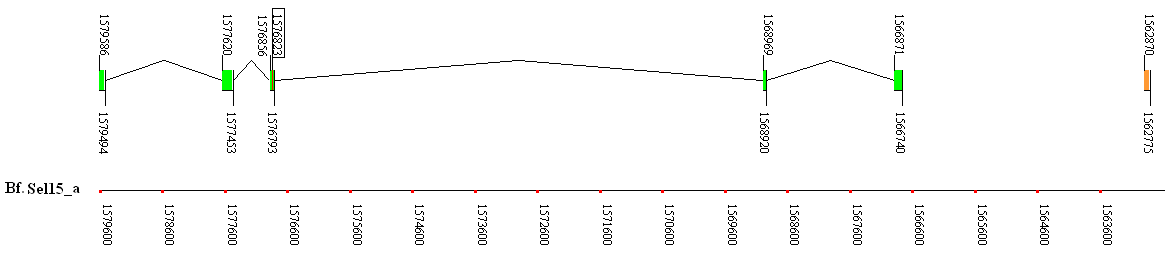

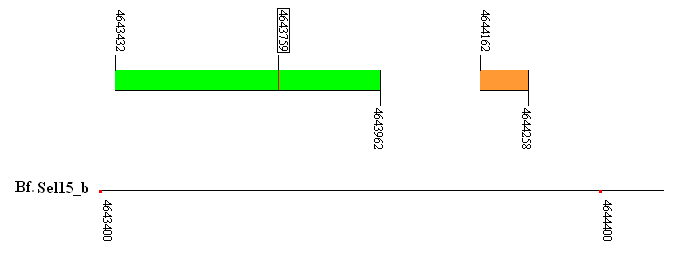


DsbA


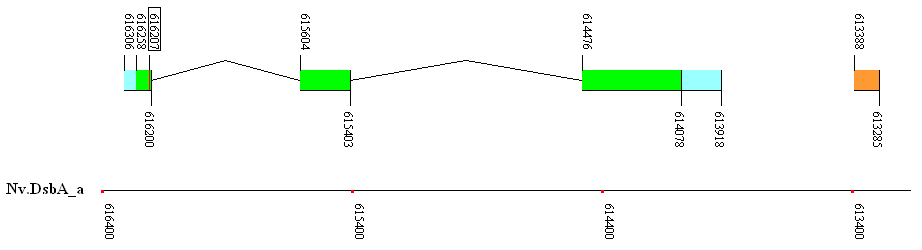


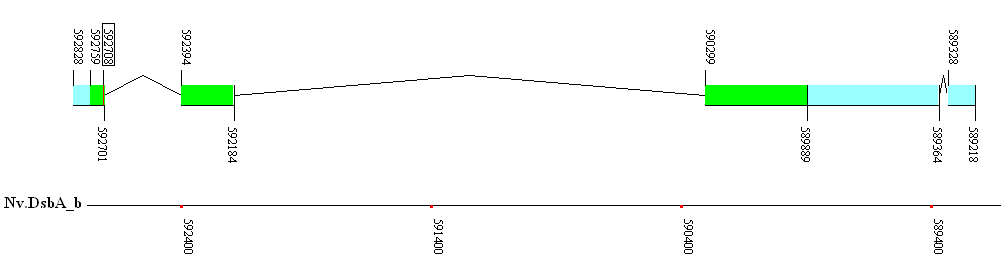


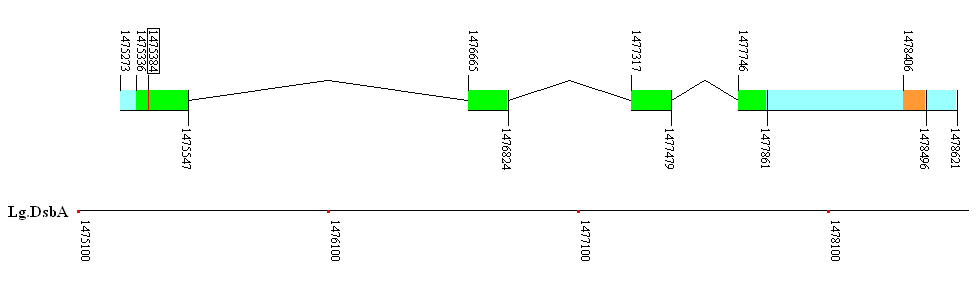

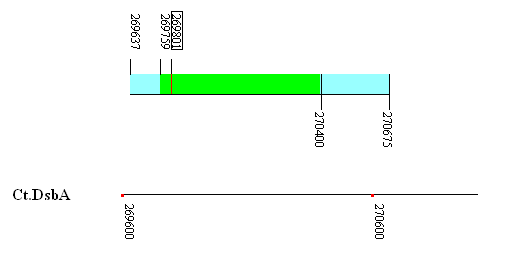

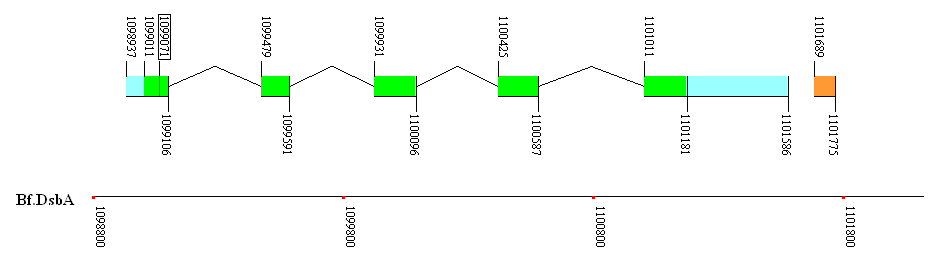


MsrA


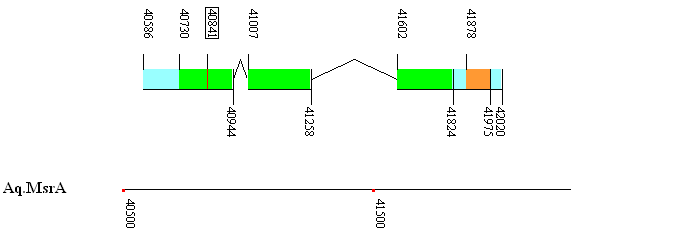


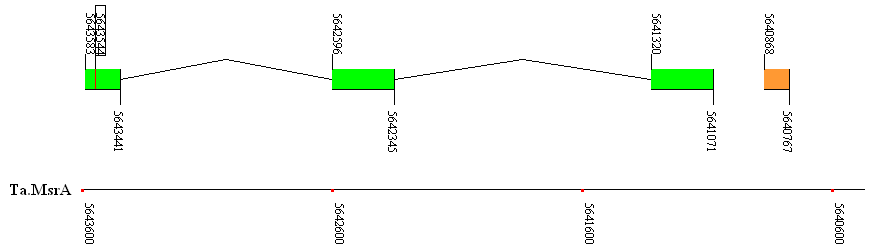

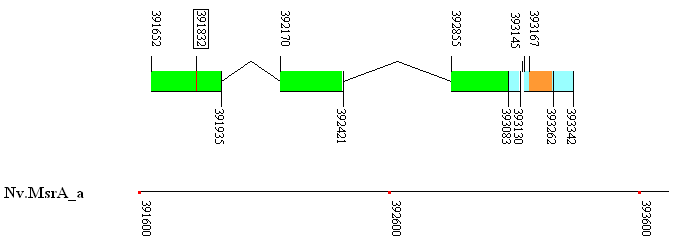

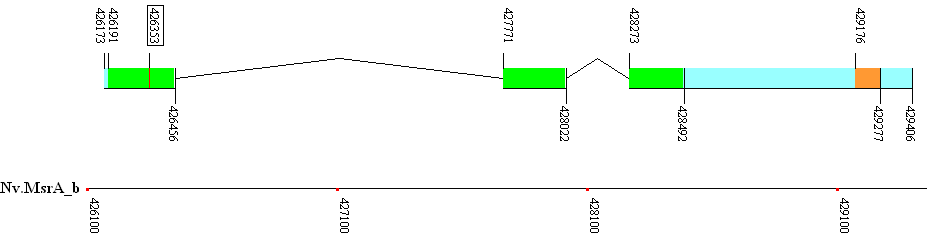

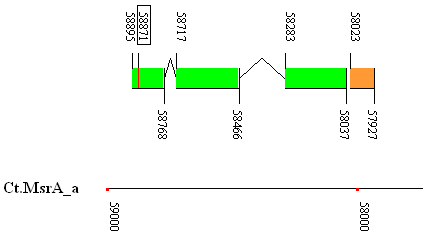

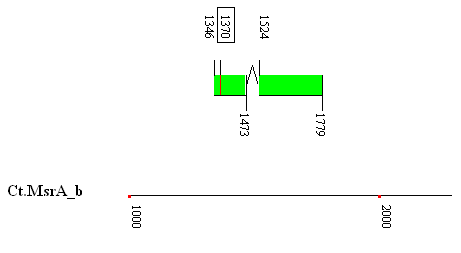


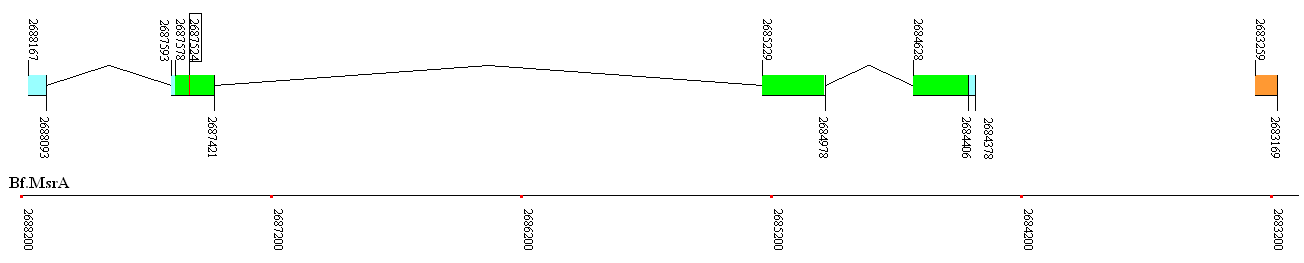


SelH


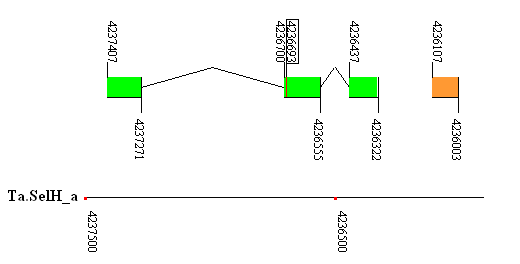

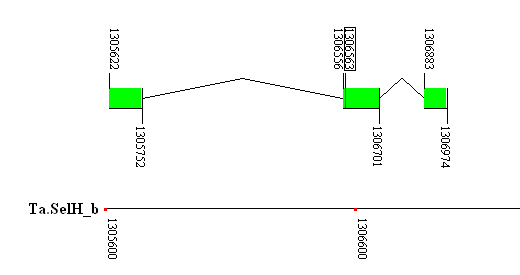


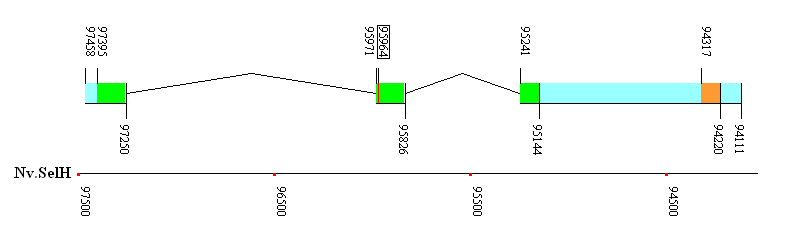

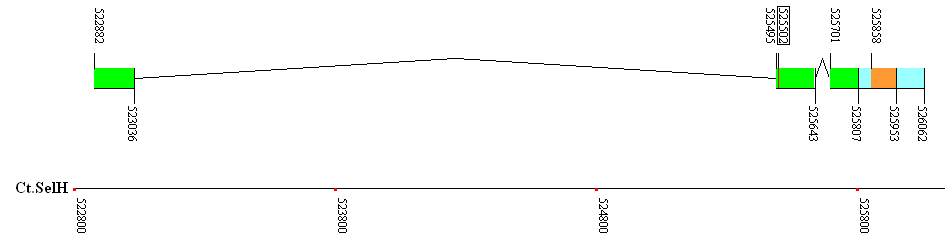

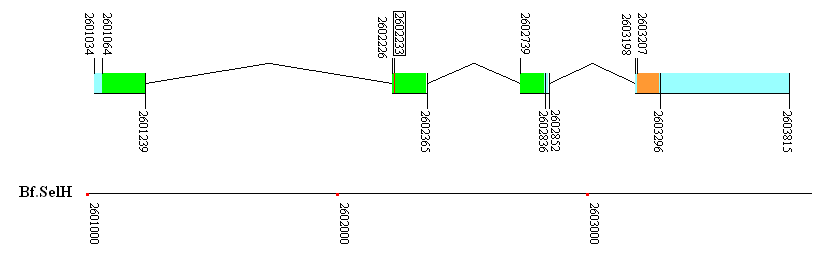


Sel:


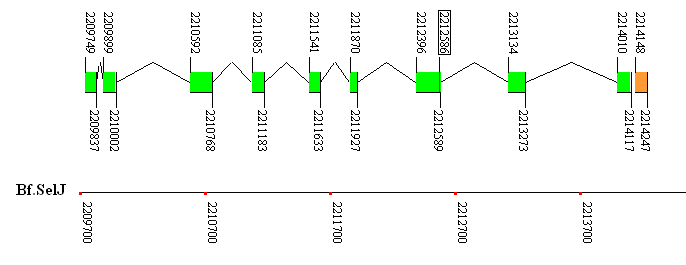


SelK


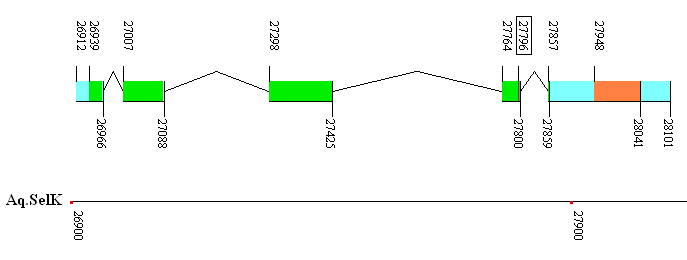

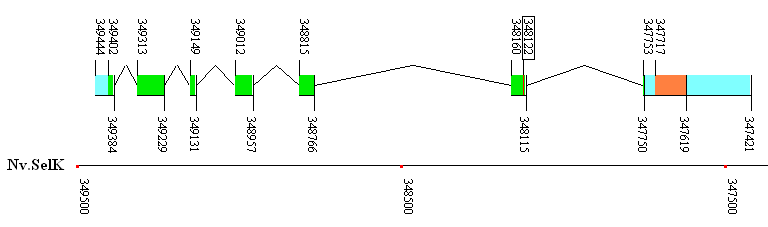

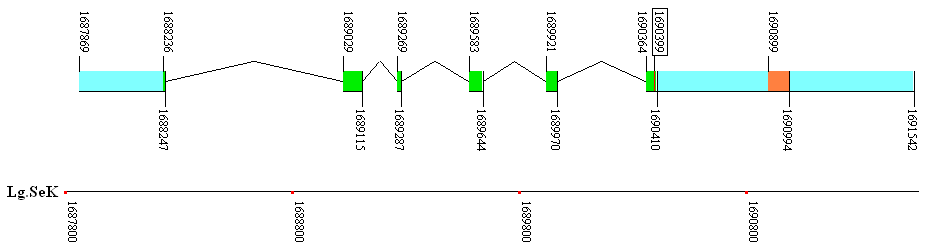

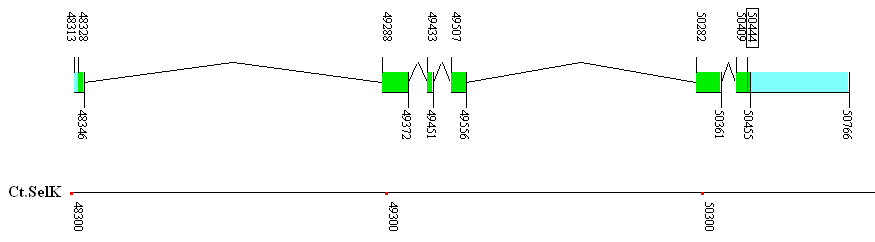


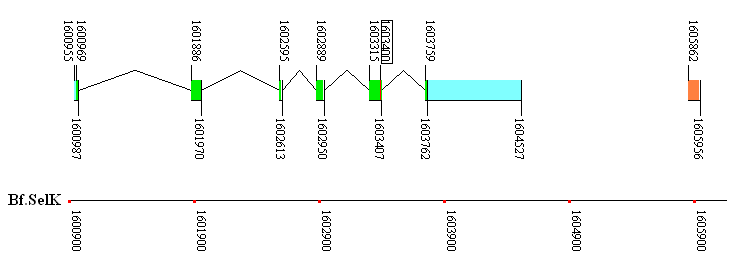


SelL


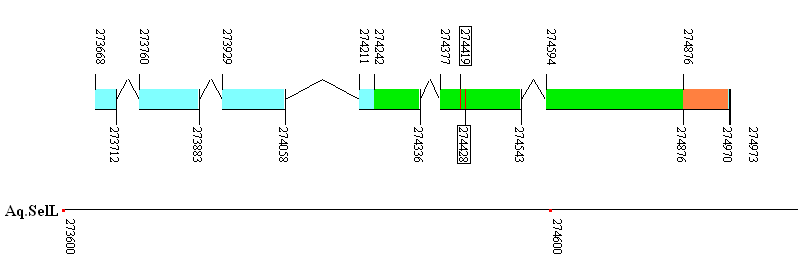


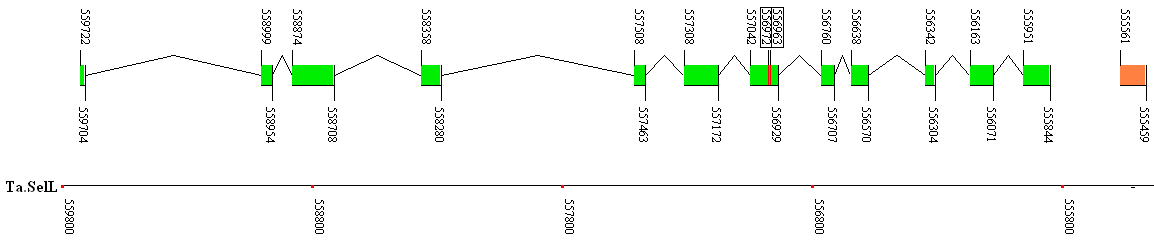

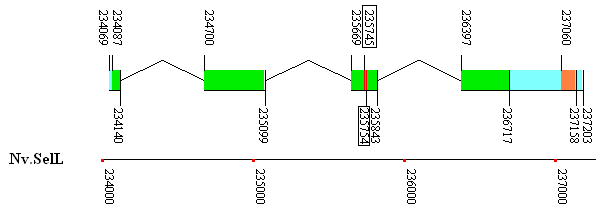


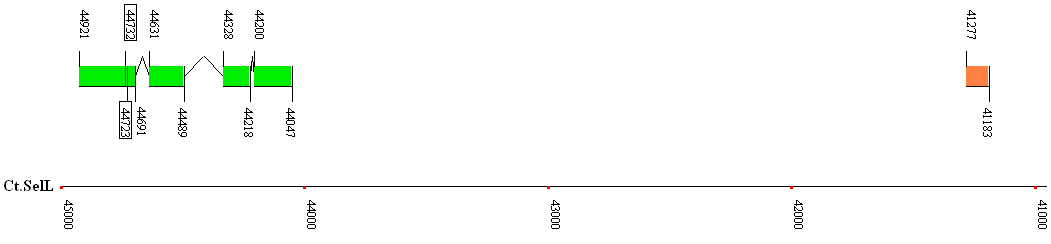

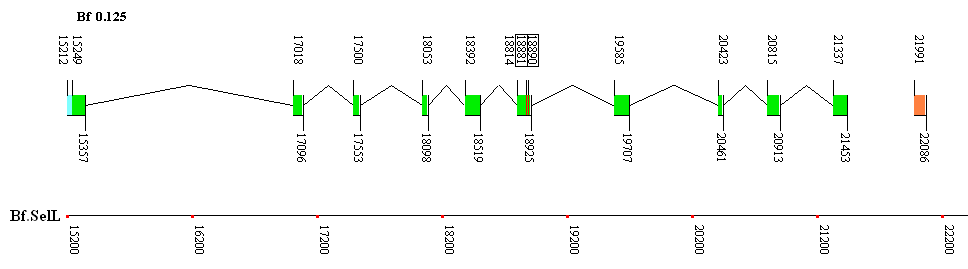


SelM


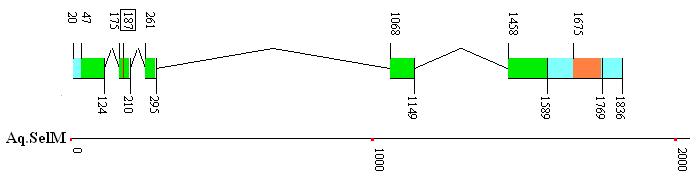


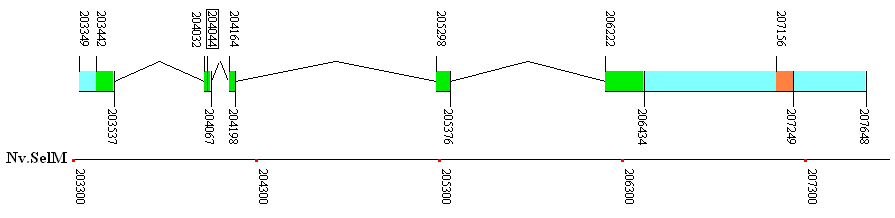

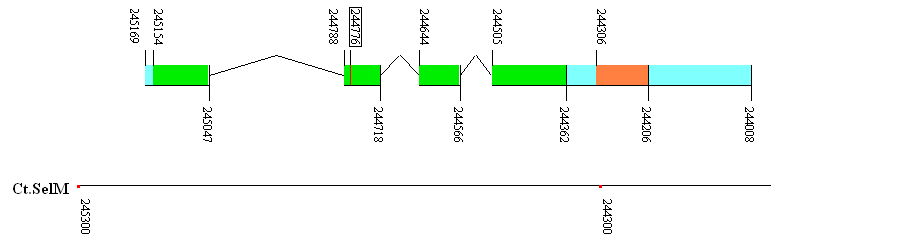

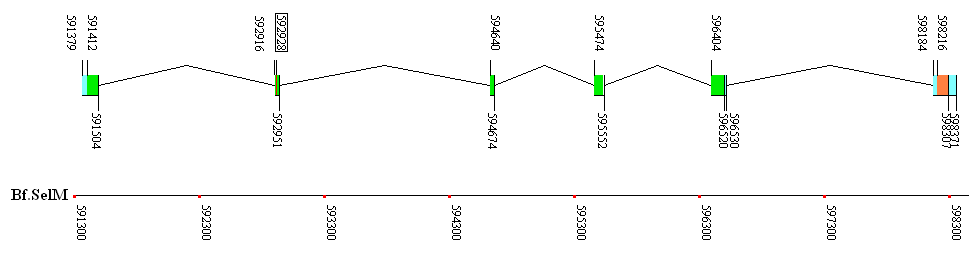


SelN


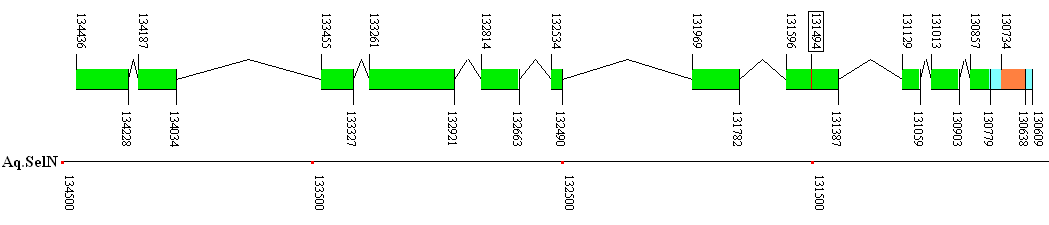


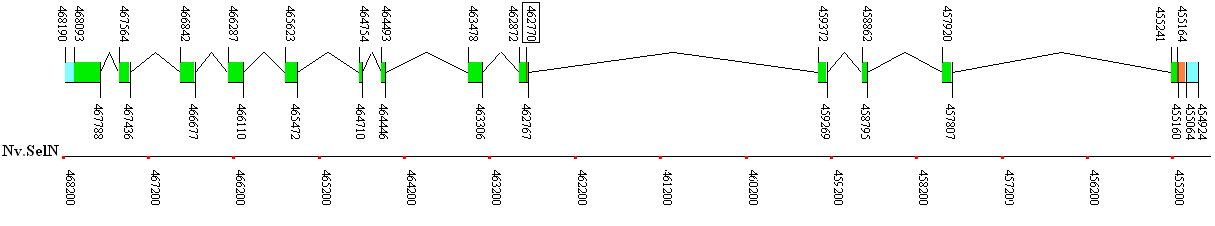


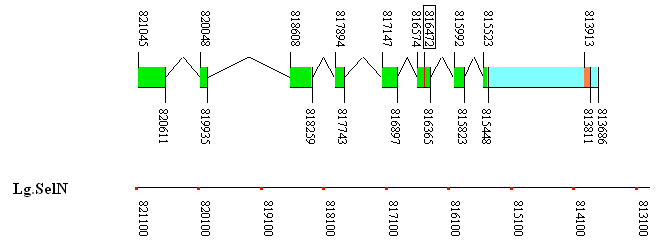


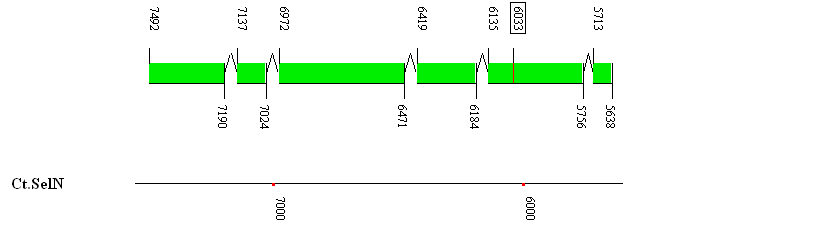


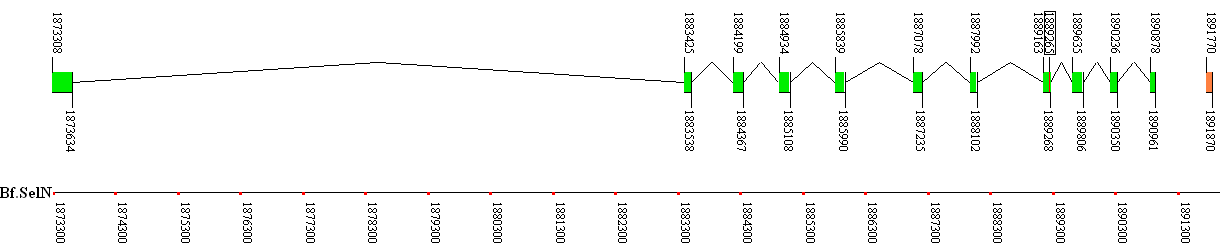


SelO


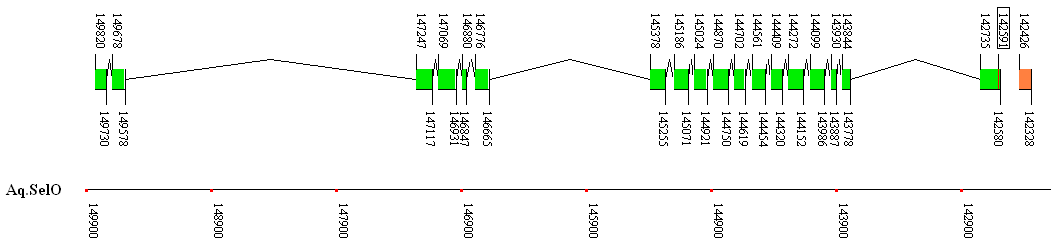

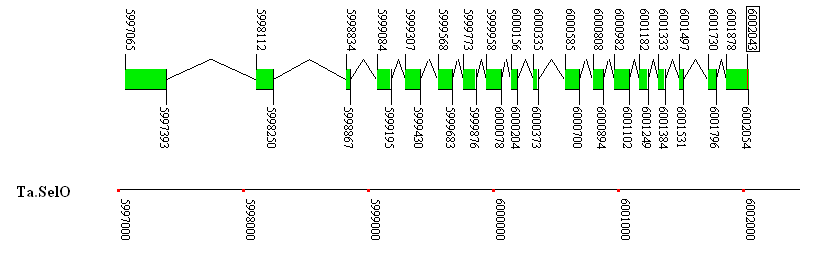


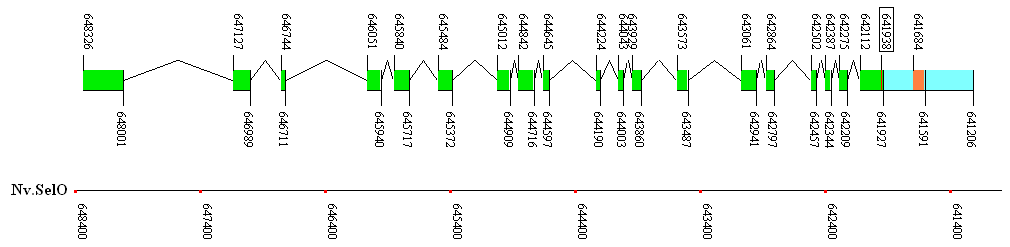


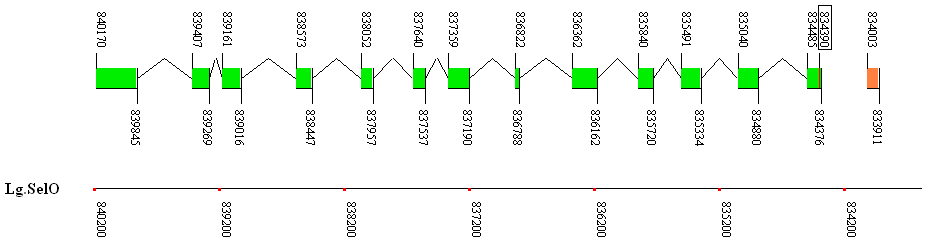


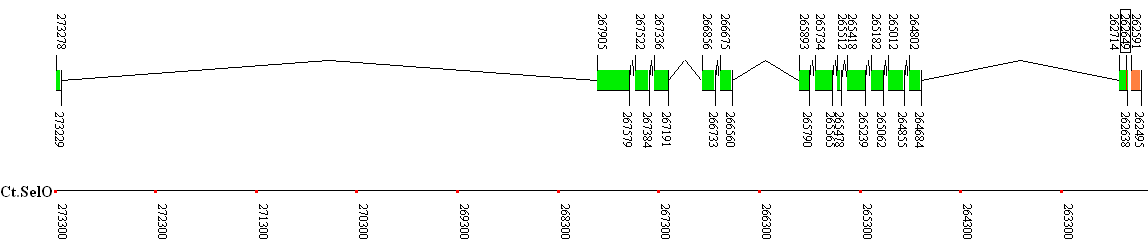

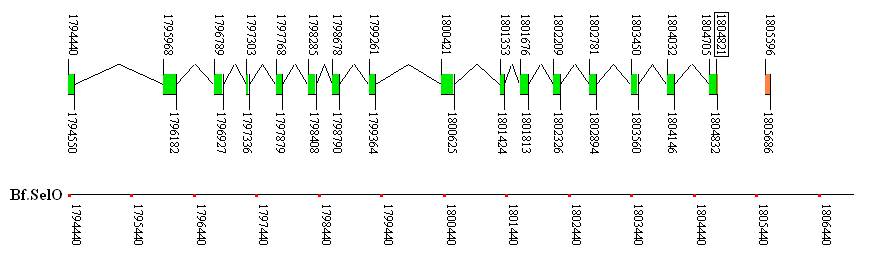


SelP:


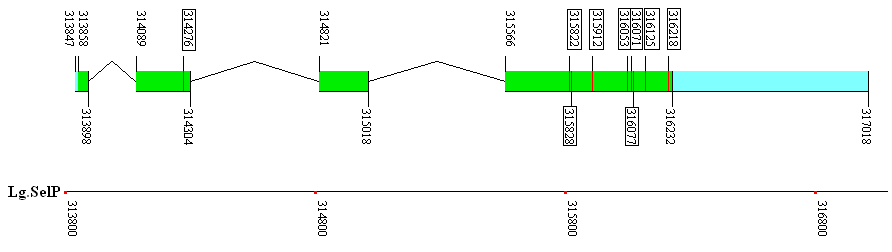


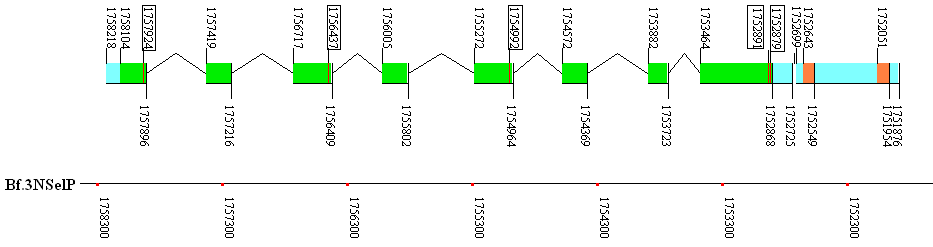


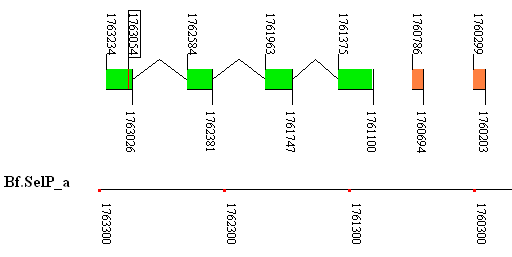


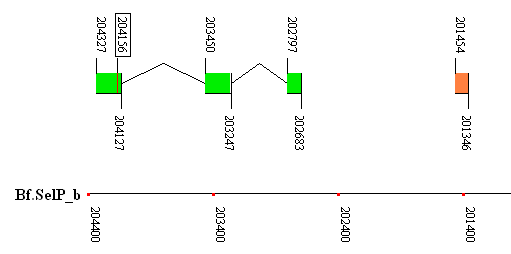

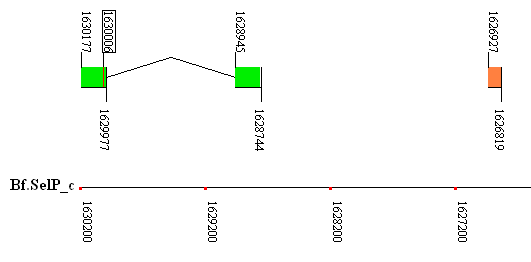


SelR


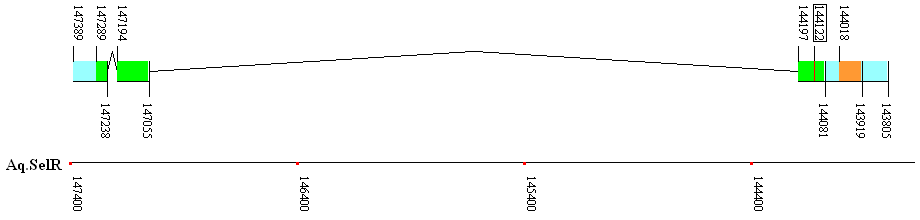


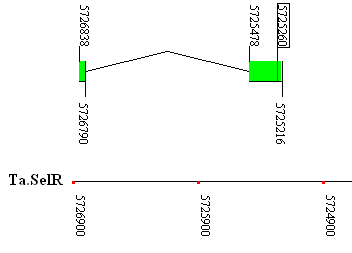

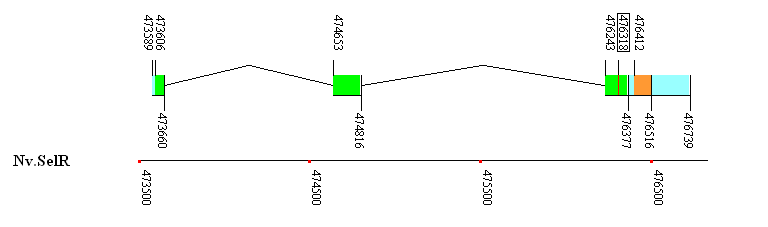


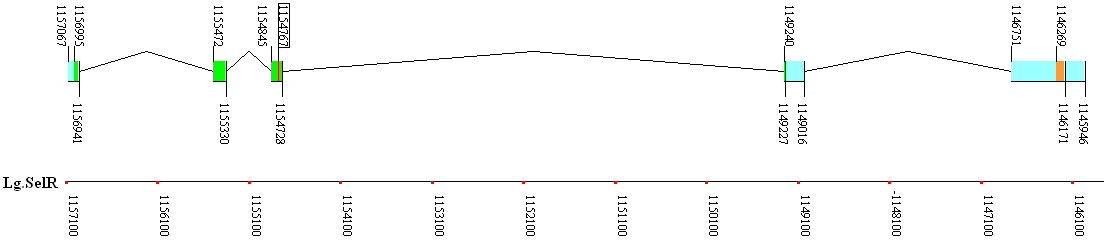


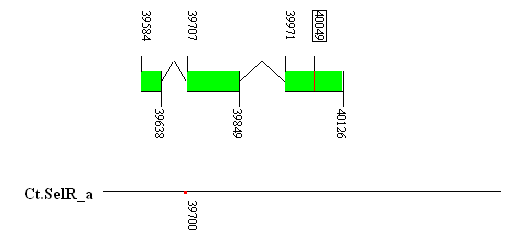

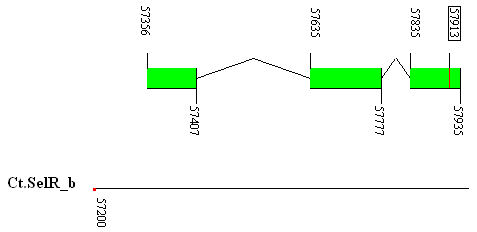


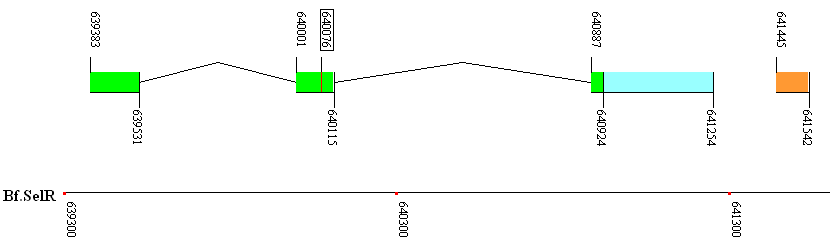


SelS


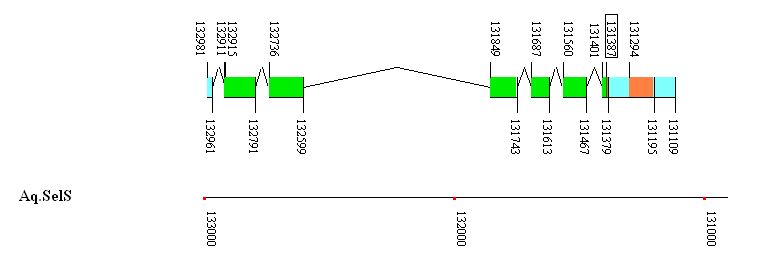

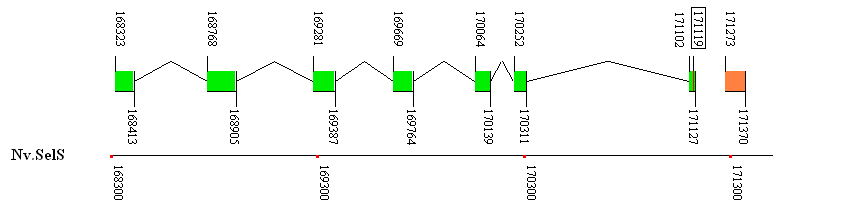

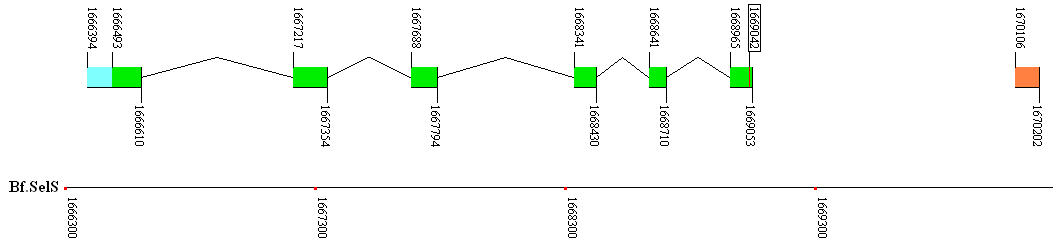


SelT


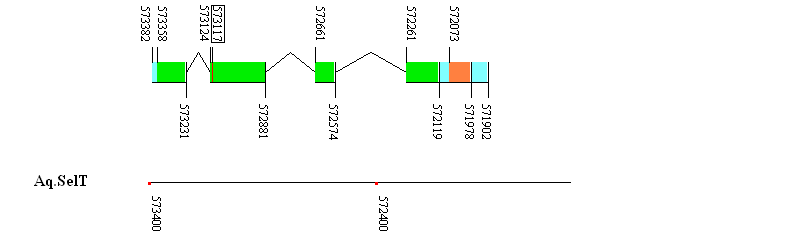


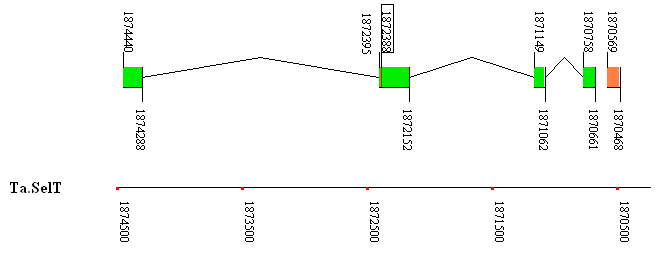


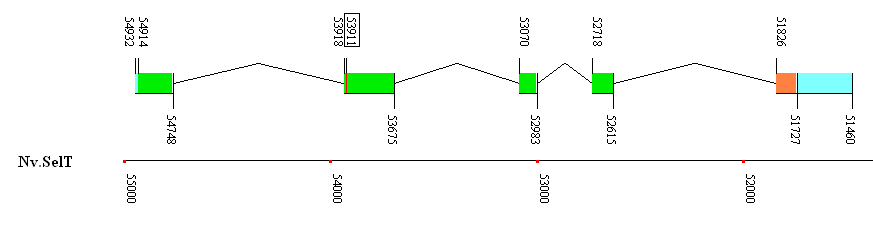


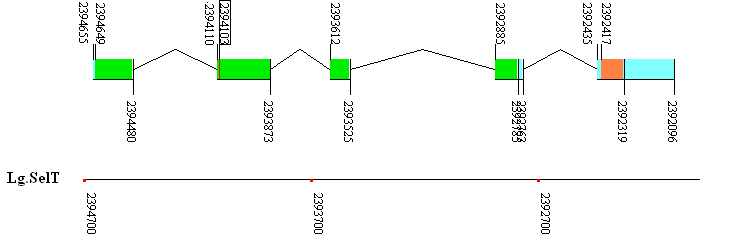


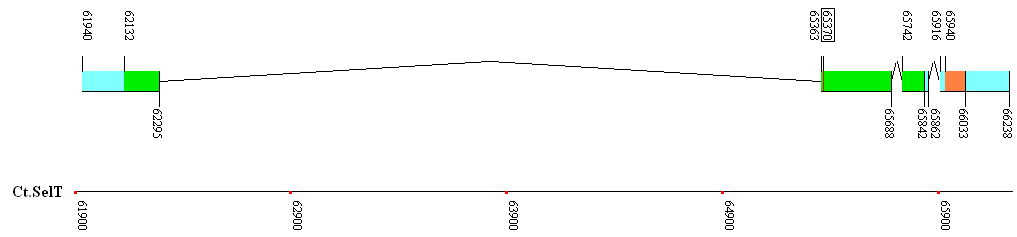

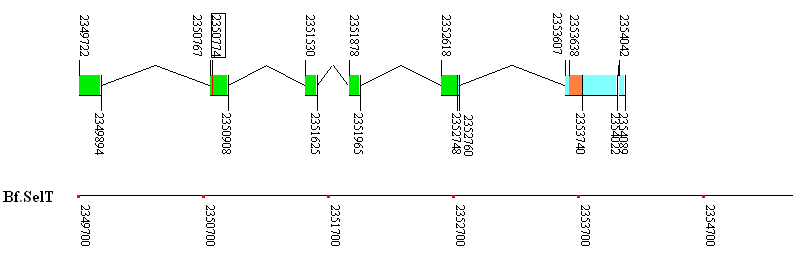


SelU1


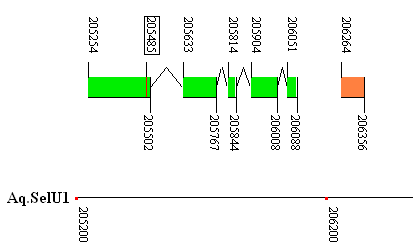

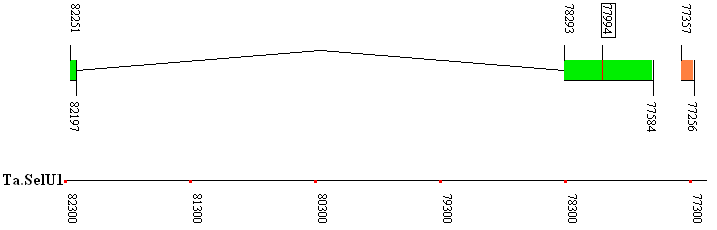


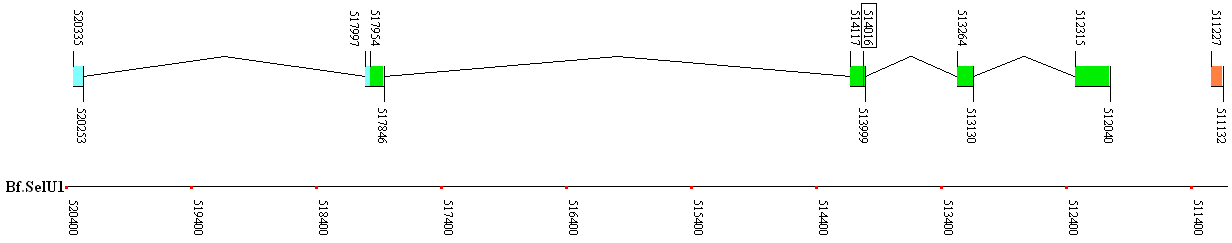


SelU:


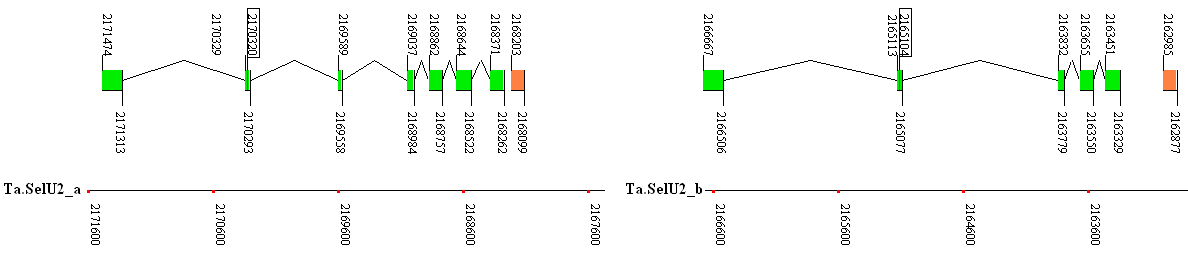


SelU3


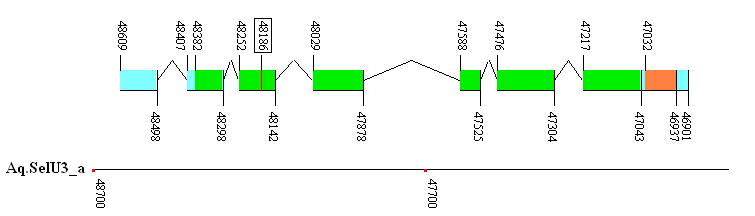


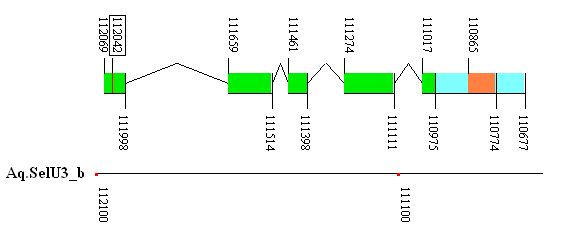


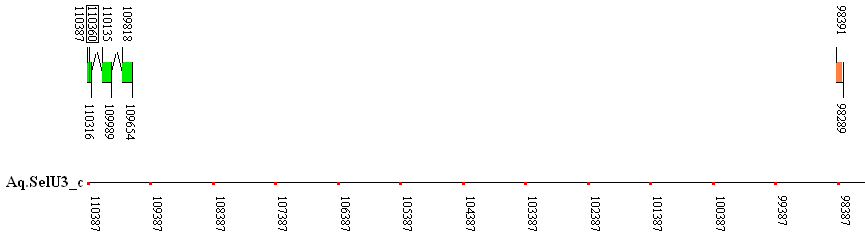


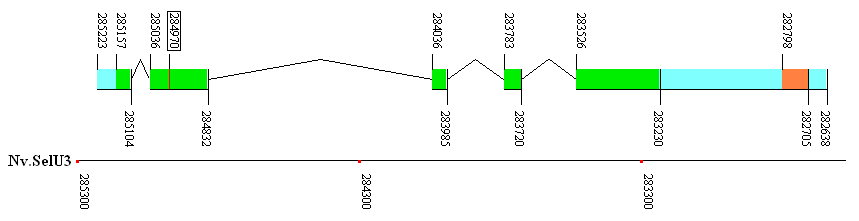


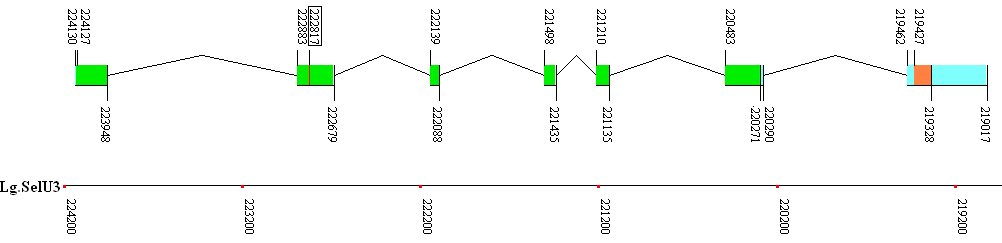

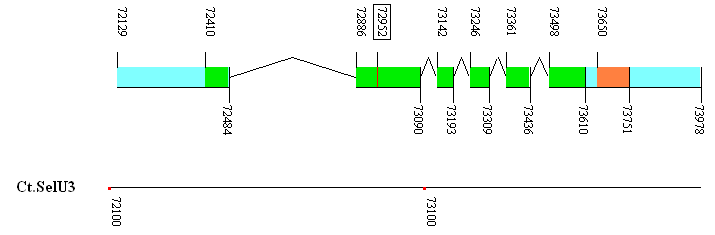


SelW


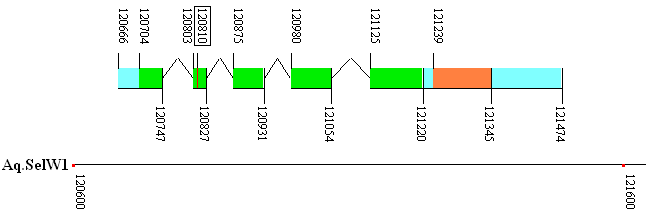


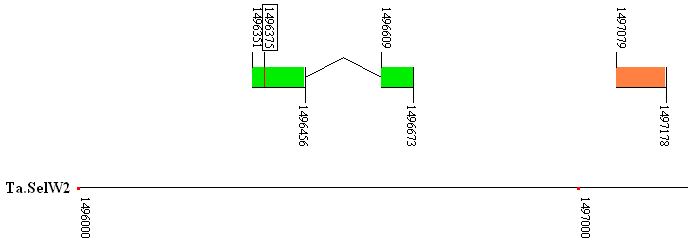


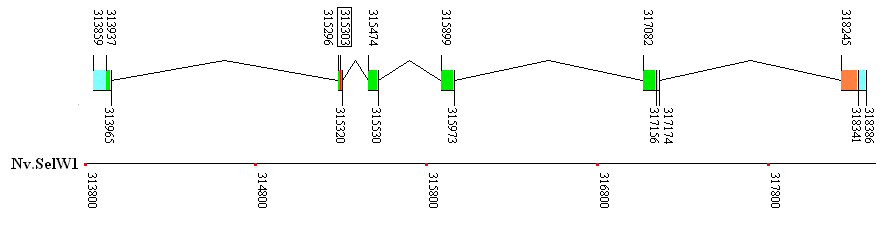


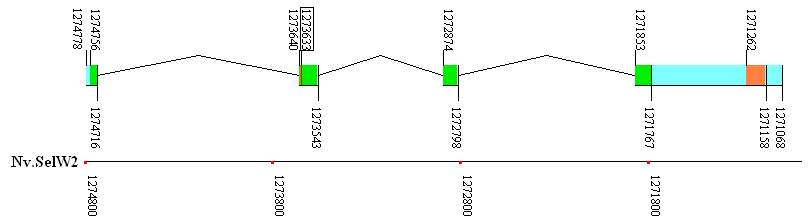


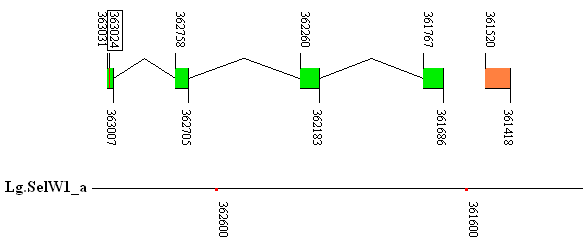


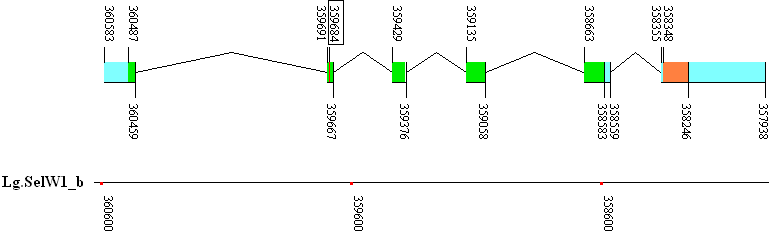


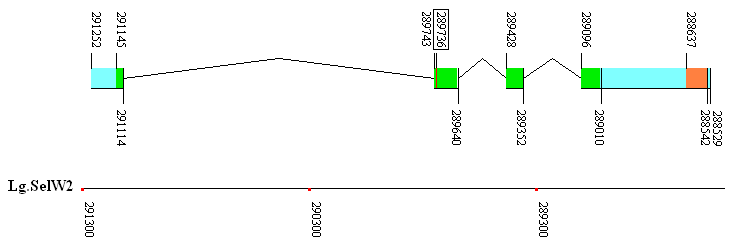


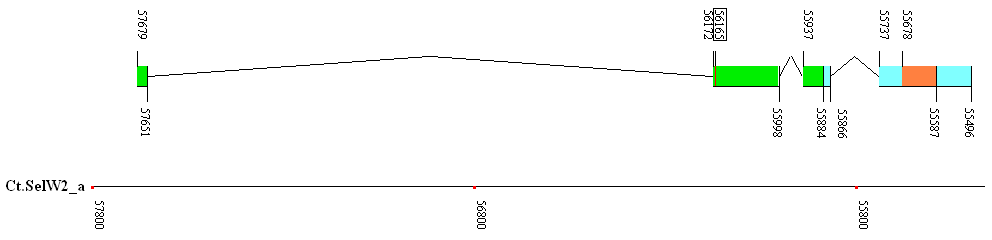


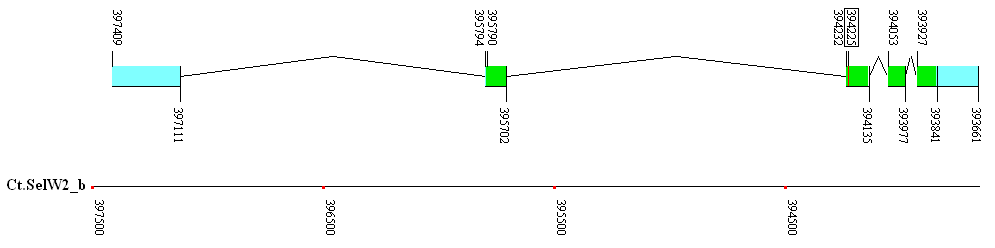


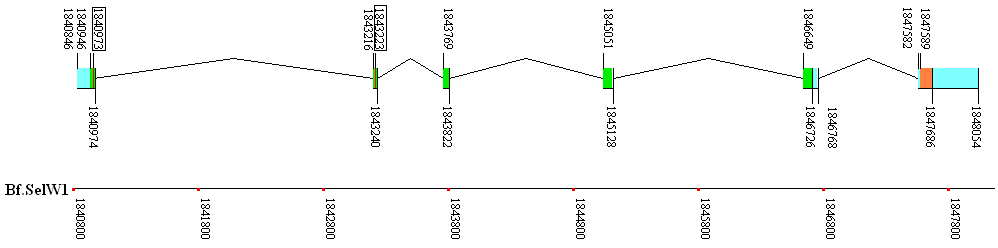


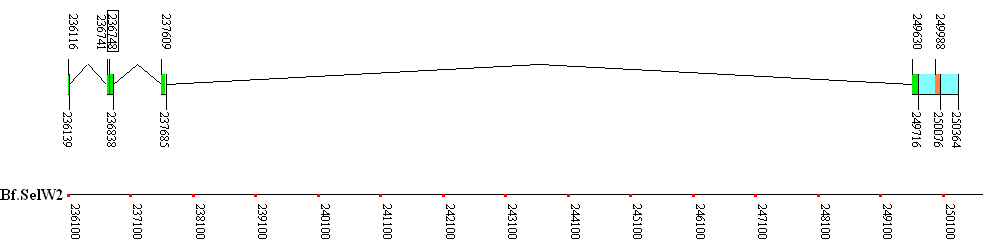


SPS


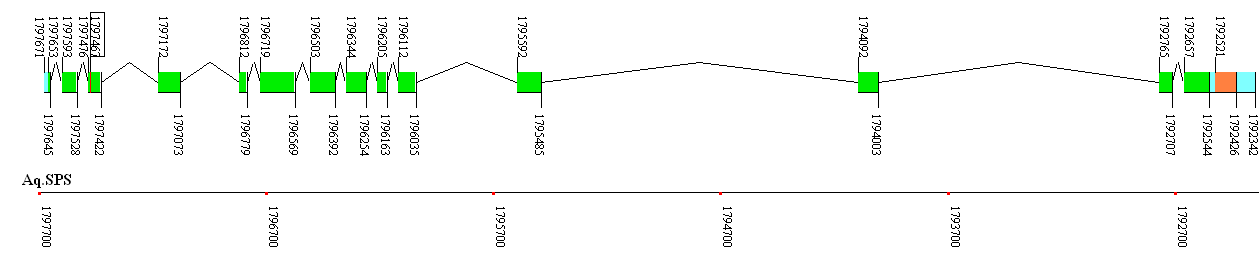


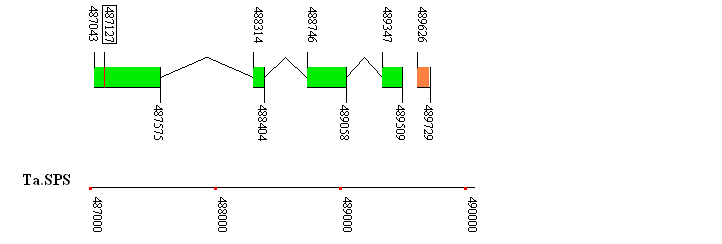


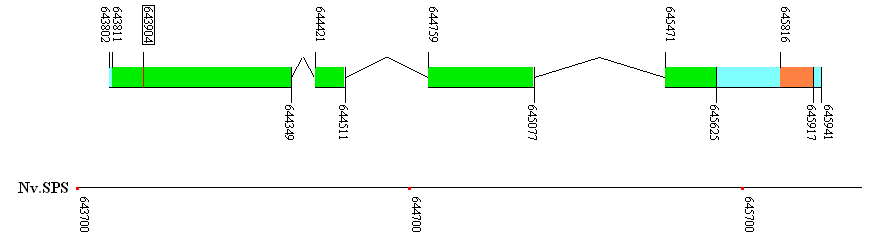


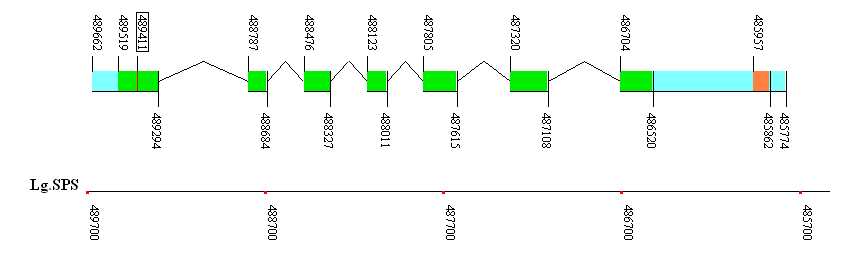


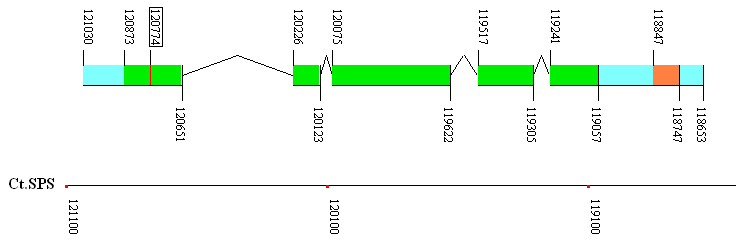


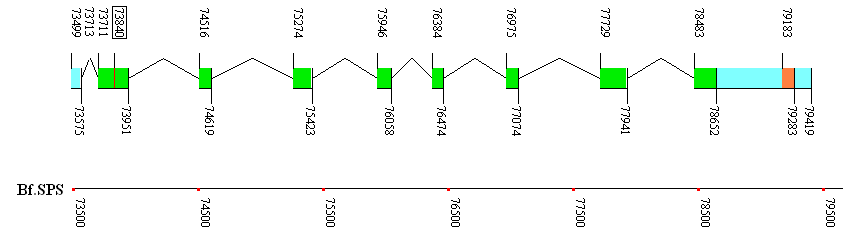


TR


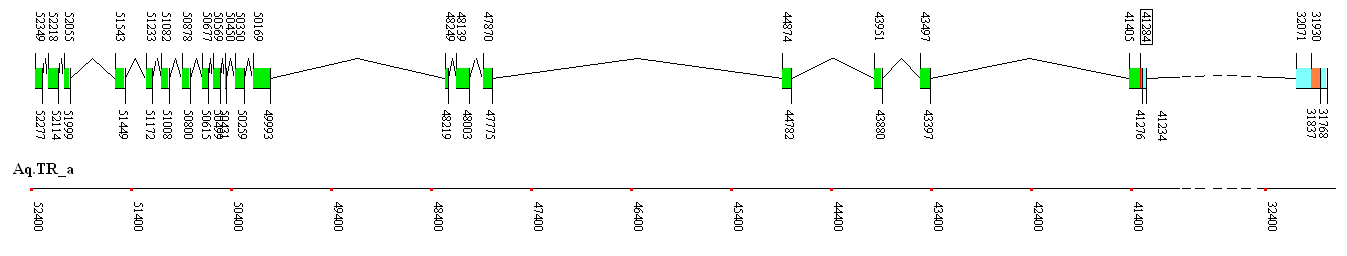


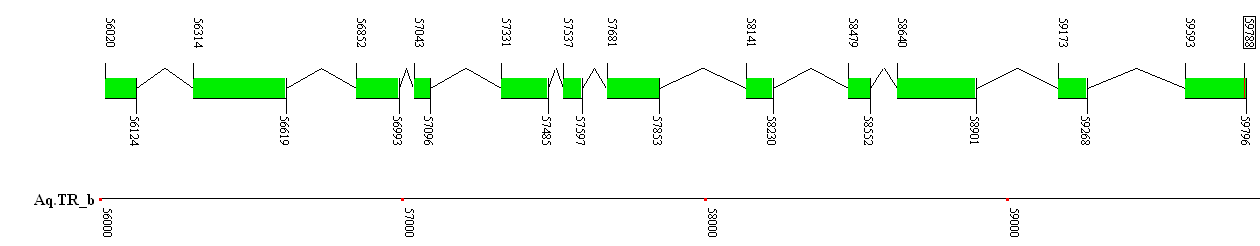


Gpx

DI

**Fig S1. Gene structures of selenoproteins identified in marine invertebrates.**

Sel15

AphC.like

MsrA

DsbA

SelH

SelJ

SelK

SelL

SelM

SelN

SelO

SelP

SelR

SelS

SelT

SelU

SelW

SPS

TR

Gpx

DI

**Fig S2. Multiple alignments of selenoproteins of marine invertebrates.**

*Amphimedon queenslandica* SECIS

*Trichoplax adhaerens* SECIS

*Nematostella vectensis* SECIS

*Lottia gigantea* SECIS

*Capitella teleta* SECIS

*Branchiostoma floridae* SECIS

*Oscarella carmela* AphC.like

**Fig S3. Secondary structures and COVE scores of the SECIS elements of marine invertebrates.**

**Fig S4. DNA sequence and Amino acid sequence of Oc.AphC.like_a.**

The Sec-TGA translated into U is highlighted by red letter U on the top of TGA. The sequence of SECIS elements is highlighted by underline.

**Fig S5. DNA sequence and Amino acid sequence of Oc.AphC.like_b.**

**Fig S6. DNA sequence and Amino acid sequence of Aq.3NSelP.**
